# Sentinel-3 Altimetry Thematic Products for Hydrology, Sea Ice and Land Ice

**DOI:** 10.1038/s41597-025-04956-3

**Published:** 2025-04-29

**Authors:** Jérémie Aublanc, Julien Renou, Fanny Piras, Karina Nielsen, Stine K. Rose, Sebastian B. Simonsen, Sara Fleury, Stefan Hendricks, Nicolas Taburet, Giovanni D’Apice, Anouk Chamayou, Pierre Féménias, Filomena Catapano, Marco Restano

**Affiliations:** 1 Collecte Localisation Satellites, 31520 Ramonville-Saint-Agne, France; 2 DTU Space, Department of Geodesy and Earth Observation, Technical University of Denmark, Kgs, Lyngby, Denmark; 3 LEGOS, Université de Toulouse, CNRS/CNES/IRD/UPS, 31400 Toulouse, France; 4 Alfred-Wegener-Institut, Helmholtz-Zentrum für Polar- und Meeresforschung, Bremerhaven, Germany; 5 European Space Agency - ESRIN, 00044 Frascati, Italy; 6 Starion, c/o ESA ESRIN, 00044 Frascati, Italy

**Keywords:** Hydrology, Cryospheric science

## Abstract

This paper presents the Copernicus Sentinel-3 level-2 altimetry Hydro-Cryo “Thematic Products”, operationally generated by ESA since September 2023. In comparison to previous product versions, the level-2 processing is now performed through three independent chains, generating three families of “Thematic Products”, for measurements acquired over hydrological, sea ice and land ice areas, respectively. Prior to the operational deployment, a full mission reprocessing was achieved with the thematic processors, providing a complete and harmonised dataset to the users. In this paper, the new architecture of the ground segment processing is presented, along with the major algorithmic developments. The main data content of the Thematic Products is also described, to indicate the key product variables for the end users. The Thematic Products have been evaluated by the Sentinel-3 Mission Performance Cluster (MPC) experts. The major results and outcomes are presented, showing the significant performance improvement achieved in comparison to previous processing versions.

## Background & Summary

Sentinel-3 is an Earth observation satellite mission developed in the frame of the European Copernicus programme. The mission’s primary objectives are to measure sea surface topography, land and sea surface temperature, and ocean-surface colour parameters^1^. The secondary objectives of the mission include, but not limited to, the delivery of geophysical products containing surface height of lakes and rivers, thickness of the floating sea ice and land ice surface elevation. To achieve these goals, the Sentinel-3 spacecraft carries four main instruments: a push-broom imaging spectrometer (OLCI), a dual view conical imaging radiometer (SLSTR), a dual-frequency SAR Radar ALtimeter (SRAL) and a Microwave Radiometer (MWR) instrument. A Precise Orbit Determination (POD) package includes a Global Navigation Satellite Systems (GNSS) instrument, a Doppler Orbit determination and Radio-positioning Integrated on Satellite (DORIS) instrument and a Laser Retro Reflector (LRR). At the time of writing, the Sentinel-3 constellation includes two satellites: Sentinel-3A and Sentinel-3B, launched respectively on 16 February 2016 and 25 April 2018. After a tandem phase of about 4 months^2^, Sentinel-3B was placed into an interleaved orbit to optimise the on-ground coverage of the constellation. The two Sentinel-3 units have a near-polar sun-synchronous orbit similar to ERS and Envisat missions. The orbit covers the polar zones up to ±81.5° in latitude, and has a revisit time of 27 days.

Sentinel-3 is the first radar altimetry mission continuously operating in Synthetic Aperture Radar (SAR) mode. In this configuration, SRAL sends radar pulses at high frequency rate (~18 kHz) to exploit the Doppler effect induced by the satellite movement^3^. This significantly reduces the along-track footprint, in comparison to conventional Low Resolution Mode (LRM). Such an enhancement is particularly valuable over continental surfaces, given their relative small-scale heterogeneities (i.e. below the radar footprint scale), in terms of surface topography, or radiometric properties, or both. The Copernicus Ground Segment (CGS) is responsible for the Sentinel-3 data downlinked by the satellites, from science core product generation (up to level-2), core products validation, to data archiving and dissemination. The European Space Agency (ESA) is responsible for the generation of Sentinel-3 Surface Topography Mission (STM) level-2 products covering the continental surfaces and the cryosphere. Whereas the European Organisation for the Exploitation of Meteorological Satellites (EUMETSAT) is responsible for the generation of Sentinel-3 STM level-2 products covering the marine surfaces, including coastal areas.

To further improve the performance of the Sentinel-3 altimetry products, ESA and the Sentinel-3 Mission Performance Cluster (MPC) developed in 2021–2022 separated delay-Doppler and level-2 processing chains dedicated to hydrology, sea ice, and land ice measurements. These “Hydro-Cryo Thematic Products” are operationally generated and distributed since September 2023, with the associated Baseline Collection n°5 (BC-005). A full mission reprocessing was accomplished prior to the operational deployment, providing a homogeneous and complete dataset to the users. The objective of this paper is firstly to present the new BC-005 Thematic Processors (section ‘Methods’), along with an overview of the Thematic Products content (section ‘Data Records’). Secondly, the MPC experts report a summary of the evaluations performed on the BC-005 Thematic Products, over hydrology, sea ice and land ice areas (section ‘Technical Validation’). For additional information, the complete evaluation of the Thematic Products, from data calibration to the estimated level-2 geophysical parameters, is available in a dedicated report^4^.

## Methods

### Overview of sentinel-3 hydro-cryo thematic processors and products

The data acquired by the payload sensors are processed on-ground by the Sentinel-3 Instrument Processing Facilities (IPF). Two IPFs have been designed for the Sentinel-3 SRAL data, to generate level-1 and level-2 altimetry products, respectively, as described thereafter.

**Fig. 1:**
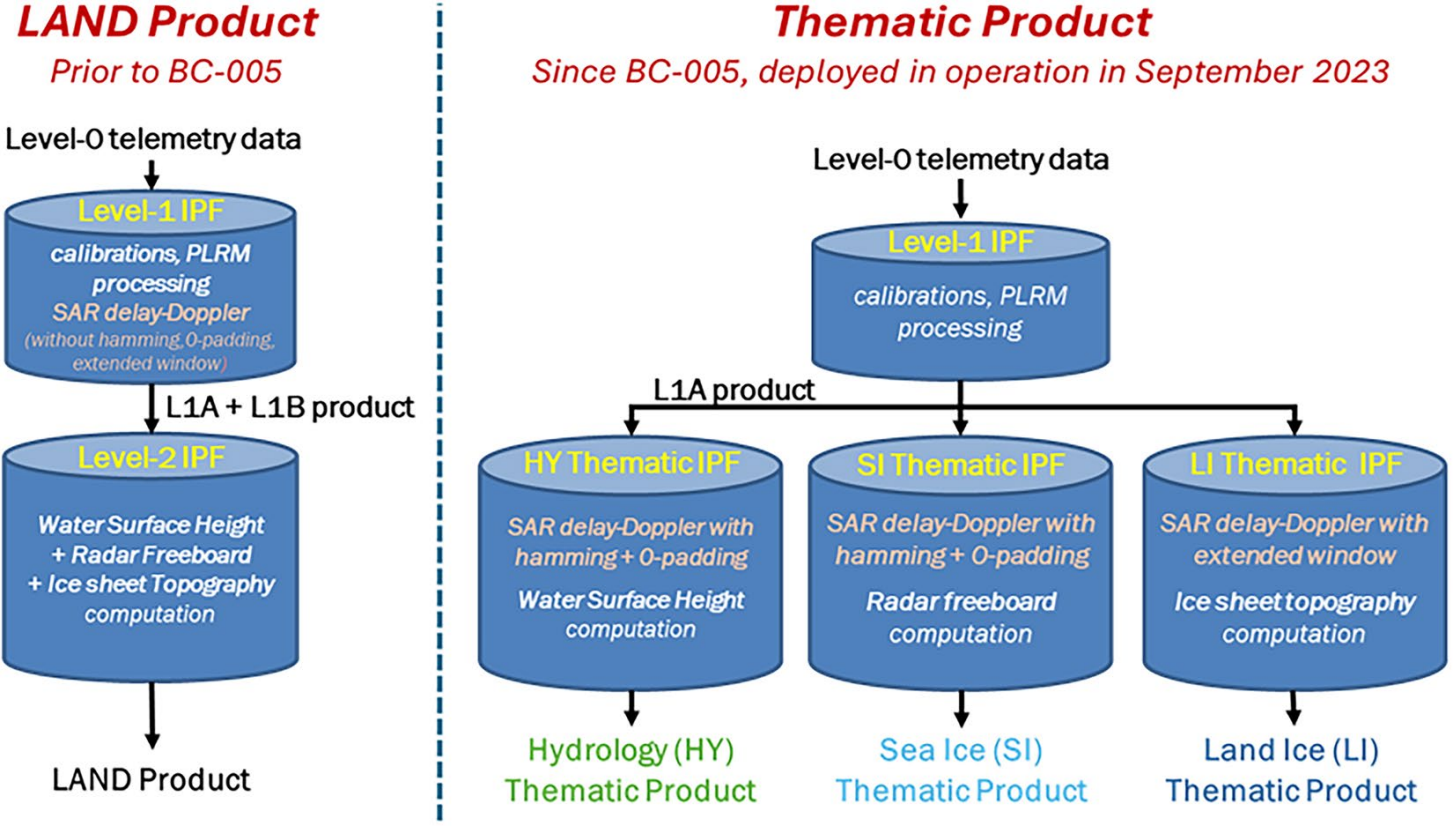
Architecture of the Sentinel-3 Hydro-Cryo Processors. On the left, the previous Sentinel-3 “Land” processing architecture, deployed in operations between 2016 and September 2023. On the right, the Sentinel-3 “Thematic” processing architecture, deployed in operations since September 2023.

In Processing Baselines (PB) prior to BC-005, the level-2 altimetry data produced by ESA were generated with a same unique IPF. The data processing was therefore identical for all the satellite measurements taken over land surfaces and sea ice areas. These level-2 Products are commonly called the “Land Products”. This terminology is kept throughout the article, when referring to the BC-005 previous versions. Since BC-005, three separated level-2 IPFs generate the so-called “Thematic Products”. As a major evolution of the BC-005, the delay-Doppler processing was moved from the level-1 IPF and integrated into the level-2 Thematic IPFs. This change enables independent adjustment of the delay-Doppler and level-2 processing chain, to accommodate for the characteristics of each thematic surface. Figure 1 displays the ground segment architecture of the Sentinel-3 SRAL Hydro-Cryo Thematic Products, including the level-1 IPF and three level-2 Thematic IPFs:

- **Level-1 IPF:** The processing starts with the level-0 telemetry data. Several calibration operations are applied to the raw data downlinked on-ground, as described in Quartly *et al*.^5^. In addition, the radar pulses are also processed disregarding the Doppler information, through a conventional LRM processing. This processing mode is named Pseudo-LRM (P-LRM). P-LRM data are useful to perform multi-mission investigations, including past & present LRM altimetry missions (e.g. TOPEX, ENVISAT, JASON-1/2/3, SARAL-AltiKa).

- **Level-2 Thematic IPFs:** Three separated level-2 processors provide three distinct Thematic Products, covering respectively hydrology, sea ice and land ice areas. The level-2 Thematic IPFs include the delay-Doppler processing to generate the SAR mode waveforms. They also contain the retracking algorithms to derive the round-trip time delay (i.e. the altimeter range) of the altimetry waveforms. Furthermore, time delay corrections are also applied to the altimeter range within the level-2 processing, to compensate for several geophysical effects affecting the radar signal. These corrections are mainly extracted from auxiliary information, as described in supplementary materials, section S1.

**Fig. 2:**
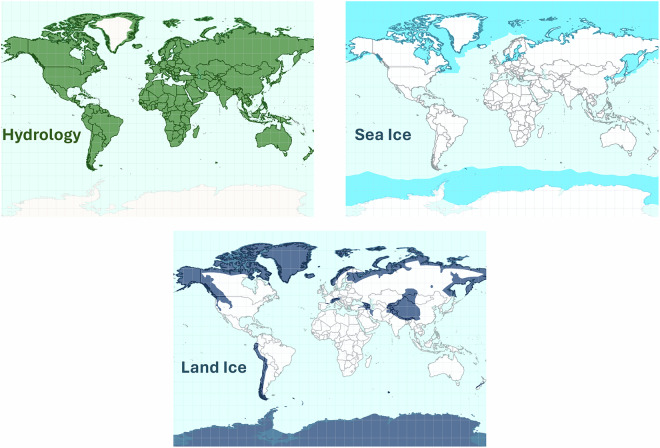
Geographical coverage of the Sentinel-3 Hydro-Cryo Thematic Products, as defined in the Thematic masks over hydrology (green, top left), sea ice (light-blue, top right) and land ice (dark-blue, bottom).

Each Sentinel-3 Hydro-Cryo Thematic Product has a dedicated geographical coverage, defined through the thematic masks reported in Fig. 2. They can be downloaded from the SentiWiki website (https://sentiwiki.copernicus.eu/web/). These spatial masks are static, except for occasional updates that can be made along the mission lifetime. For the Hydrology Thematic Product, the mask coverage includes all the continental surfaces, except for the Antarctic and Greenland ice sheet interiors. For the Sea Ice Thematic Product, the mask is defined based on the maximum of sea ice extent, according to sea ice climatology from National Snow and Ice Data Center (NSIDC)^6,7^. For the Land Ice Thematic Product, the mask includes the Antarctica and Greenland ice sheets, along with glacier areas as defined in the Randolph Glacier Inventory database^8^.

The main changes implemented in the BC-005 Thematic IPFs are in the delay-Doppler processing algorithms, as described in section ‘Delay-Doppler processing’ No major evolutions were applied to the other level-2 algorithms. A summary description of the three Thematic level-2 processing is available in section ‘Surface topography retrieval’ In supplement to data evaluation, this publication is meant to provide an overview of the Sentinel-3 Thematic Products and inform users on the key elements of the ground segment processing and product content. Some of the aspects reported in this section are also available in a dedicated Product Handbook^9^, which also contains  supplementary information related to the Sentinel-3 Hydro-Cryo Thematic Products. The IPFs algorithms are also described in Algorithm Theoretical Basis Documents^10^.

### Delay-doppler processing

When operating in its nominal “SAR mode”, SRAL continuously transmits bursts of radar pulses at a frequency rate of about 80 Hz. The radar bursts contain 64 Ku-band and 2 C-band pulses, to implement a dual-frequency ionosphere propagation correction over the ocean. Within a burst, the Pulse Repetition Frequency (PRF) is approximately 18 kHz. By exploiting the Doppler effect caused by the satellite movement, the 64 Ku-band coherent pulses can be processed to split the surface on 64 contiguous Doppler strips, perpendicular to the satellite’s direction of motion. Considering the Sentinel-3 orbital and instrumental characteristics, the 64 contiguous delay-Doppler beams have a thin strip-shaped footprint, of about 330 m in the along-track direction, and 15 km in the cross-track direction (by considering the area sampled by the entire SAR mode waveform, in the case of a flat surface).

The UnFocused-Synthetic Aperture Radar (UF-SAR) processing implemented in the Sentinel-3 IPF follows the general concept from Raney^3^. In summary, sample locations are firstly defined along the satellite track at a posting rate of ~20 Hz, corresponding to an along-track distance of about 330 m on-ground. This along-track distance is calculated to match with the UF-SAR along-track footprint resolution, ensuring minimum footprint overlap between two consecutives 20 Hz SAR mode measurements. For each sample locations, delay-Doppler beams produced from different radar bursts are gathered to generate a delay-Doppler stack. Each 20 Hz delay-Doppler stack nominally includes 180 single-looks acquired at different viewing angles along the satellite track. After range migration corrections, the stacked signals are multi-looked (i.e., summed) to generate the SAR mode waveform, which is the input to level-2 retracking algorithms.

In the Hydrology and Sea Ice Thematic IPFs, the delay-Doppler processing was upgraded with two features. Firstly, the SAR mode waveforms are oversampled in the range window by a factor of two, using a zero-padding technique. The zero-padding is applied to the complex single-looks signals of the delay-Doppler stack, prior to the range compression^11^. Therefore, the SAR mode waveforms generated by the Hydrology and Sea Ice Thematic IPFs contain 256 range gates (compared to 128 range gates for the previous PB). This is valuable for refining the range sampling of peaky waveforms measured over specular surfaces. With a standard UF-SAR processing, such waveforms may be sampled only over two or three range gates, which is insufficient to derive the surface topography with accuracy^12^. Secondly, a Hamming weighting window is applied to the 64 pulses included in a radar burst, in the azimuth dimension, and prior to the azimuth Fourier Transform. The Hamming window decreases the energy of the azimuth impulse response side-lobes. For instance, the first side-lobe energy is lowered from approximately -13 dB to -42 dB. The goal is to remove spurious energy spreading in the azimuth direction in the delay-Doppler map. The level of this unwanted energy can be high in cases of specular return, and can contaminate the SAR mode waveform^13^. The effects of zero-padding and Hamming window in the SAR mode waveforms generated over sea ice are illustrated in supplementary materials, section S2.

In the Land Ice Thematic IPF, the delay-Doppler processing was upgraded with the so-called “extended window processing”^14^. This technique optimises the generation of SAR mode waveforms over the ice sheet margins. Over these areas, the on-board tracking command can significantly vary during the ~two seconds of UF-SAR multi-looking. Hence, after range migration operations, energy sampled in the single-look waveforms can be moved out of the 128-sample window. To compensate for this effect, the range window of the delay-Doppler stack is artificially extended prior to the range migration operations, from 128 to 512 samples. With this method, additional backscattered energy remains kept in the delay-Doppler stack. After multi-looking operation, the SAR mode waveform is re-centred within a conventional 128-sample window analysis, using an Offset Centre Of Gravity (OCOG/ICE-1) retracker^15^. The effect of the extended window processing in the SAR mode waveforms is illustrated in supplementary materials, section S3.

### Surface topography retrieval

The main objective of radar altimetry is to determine the Earth’s surface topography beneath the satellite. This requires the knowledge of three key components:

1-Determining the satellite location at the time of the measurement

2-Estimating the two-way travel time of the transmitted electromagnetic pulses, from the satellite to the Earth’s surface

3-Correcting for different geophysical effects affecting the altimeter range

This simplifies into the general equation:

$${surface\; height}={satellite\; altitude}-{altimeter\; range}+\sum \,{geophysical\; corrections}$$

The altitude of the Sentinel-3 satellite is estimated using the on-board GNSS and DORIS instruments and operationally provided by the Centre National d’Etudes Spatiales (CNES) and the Copernicus POD service to the Sentinel-3 CGS. Several geophysical corrections are calculated to compensate for delays induced by the atmosphere, and for tidal effects. These geophysical corrections are described for each of the three thematic surfaces in supplementary materials, section S1. The altimeter range is derived from the radar waveforms by means of a so-called “retracking” algorithm. In the Thematic IPFs, different retrackers are employed depending on the nature of the surface, as explained hereafter.

- **Hydrology Thematic Product**
  The main geophysical parameter estimated from altimetry measurements acquired over hydrological surfaces is the Water Surface Height (WSH). In the Hydrology Thematic Product, it is available at 20 Hz rate within the variable “***elevation_ocog_20_ku***”, referenced to the WGS-84 ellipsoid. The OCOG/ICE-1 retracking algorithm^15^, with an 80% threshold, is implemented to derive the altimeter range from the SAR mode waveforms. In addition, users have also access to the altimeter range estimated from the initial version of the SAMOSA retracker^16^. It is recommended to make use of SAMOSA outputs only for measurements acquired over large lakes, or enclosed seas, taken at least ~8–10 km away from the land surface. In fact, the retracking algorithm was developed for ocean radar altimetry, and the modelling may become inaccurate in the event of land area sampled within the radar footprint.
  It must be noted that Sentinel-3 is operating with Open Loop Tracking Commands (OLTC) over Inland Waters. This choice was made to optimise the radar signal acquisition over identified water bodies^17,18^. At the time of writing, about 148 000 hydrological targets are monitored in open loop tracking mode, for the two Sentinel-3 units. Sentinel-3 is also operating with OLTC over ocean and sea ice areas. A conventional closed loop tracking mode is used over the Antarctic and Greenland ice sheets.
- **Land Ice Thematic Product**
  The main geophysical parameter estimated from altimetry measurements acquired over land ice is the surface elevation. In the Land Ice Thematic Product, it is available at a 20 Hz rate in the variable *“****elevation_ocog_20_ku****”*, referenced to the WGS-84 ellipsoid. As for the Hydrology Thematic IPF, the altimeter range is derived from the SAR mode waveforms with the OCOG/ICE-1 retracking algorithm^15^, with an 80% threshold.
  As a major difference with hydrology measurements, the surface elevation is not estimated at nadir, but relocated at the so-called Point Of Closest Approach (POCA). The POCA coordinates are available within the variables “***lon_cor_20_ku***” and “***lat_cor_20_ku***”. The relocation method implemented in the Sentinel-3 IPF assumes a linear slope at the footprint scale to determine the POCA. The surface slope is extracted from slope models, which are derived from auxiliary Digital Elevation Models (DEM)^19^. Prior to BC-005, the slope models were generated using the so-called “RAMP DEM” for Greenland^20^, and Bamber *et al*. DEM^21^ for Antarctica. The relocation algorithm was initially developed for LRM altimetry and is described in the Envisat RA2/MWR Product Handbook^22^. It was adapted to the geometry of delay-Doppler altimetry for usage in the Sentinel-3 ground segment.
  In the BC-005 version, and previous ones, the relocation processing is performed for measurements acquired over the Antarctic and Greenland ice sheets. Therefore, it is advisable to use the surface topography variables of the Land Ice Thematic Products only over these areas. For other land ice regions, expert users have access to all parameters (e.g. radar waveforms, geophysical corrections) to retrieve the surface topography with their own level-2 processing chain.
- **Sea Ice Thematic Product**
  The main geophysical parameter estimated over sea ice by the ground segment processing (in BC-005 version) is the radar freeboard, defined as the altimetry-derived elevation of the ice surface above local sea level. The local sea surface height is interpolated all along the trajectory from measurements of heights in the fractures in the ice, or “leads”. The radar freeboard is the difference between the height above the floes and this sea level. It is worth noting that this radar freeboard is not directly the freeboard of the ice, as the Ku-band radar wave penetrates the snow medium.

In the Sea Ice Thematic Product, radar freeboard is available at 20 Hz rate within the variable “*freeboard_20_ku*”. The freeboard processing chain implemented in the Sea Ice Thematic IPF follows the main operations described in Tilling *et al*.^23^:

- **Surface type discrimination**: the surface sampled by the altimeter is classified as “leads” or “sea ice” or “open ocean” or “unclassified”. The classification is achieved using the SAR mode waveform peakiness^24^, and sea ice concentration at the measurement location. Since the new BC-005, the sea ice concentration is retrieved from the OSI-SAF-430 product^25^.
- **Surface topography estimation**: For specular echoes acquired over leads, the altimeter range is derived from the SAR mode waveforms with the Giles retracking method^26^. For diffuse echoes over open ocean and sea ice floes, a 70% threshold retracker is applied^23^. The surface topography is referenced to a Mean Sea Surface (MSS), and is therefore defined as a “surface height anomaly”. The DTU21 model^27^ is used as the reference MSS.
- **Interpolation of lead heights and radar freeboard computation**: the sea surface height anomaly is interpolated along the track, at the locations of the measurements classified as “sea ice”. The radar freeboard is computed for records identified as “sea ice” only. It is calculated as the difference between the surface height anomaly of sea ice floes, and the interpolated surface height anomaly of leads.

### Recent and upcoming data processing improvements

Now that the ground segment processing is autonomous for the three thematic surfaces, the algorithms and products can be more efficiently tailored to address the needs of the data users. In the PB 3.29, operationally deployed in July 2024, the content of the Thematic Products has been improved with new product variables.

For the Hydrology Thematic Product, with a flag providing information related to the OLTC settings. For the Sea Ice Thematic Product, with a “sea ice type” information, interpolated from OSI-SAF 403 d product^28^. For the Land Ice Thematic Product, with a surface type information, interpolated from BedMachine dataset^29^. In the medium term, alternative retracking solutions are envisaged to further improve the performance in the estimated water level, provided in the Hydrology Thematic Product. Furthermore, operational delivery of sea ice thickness within the Sea Ice Thematic Product is scheduled by 2025. For the ice sheet topography, the highest priority is to improve the performance over the margins of the continents. As a first step, ESA plans to provide Sentinel-3 demonstration products for the users, generated with the so-called AMPLI processing chain^30^. This data set is planned to be released in early 2025. Moreover, another top priority is to assess and correct for the snow volume scattering, impacting the ice sheet topography estimated by Sentinel-3.

## Data Records

The “Sentinel-3 Level-2 Hydro-Cryo Thematic Products, BC-005”^31^ are freely available in the ESA Copernicus Data Space Ecosystem (referenced as “*LAN_HY*”, “*LAN_SI*” and “*LAN_LI*”, for Hydrology, Sea Ice and Land Ice Thematic Products, respectively). The data set Digital Object Identifier (DOI), and associated landing page, is provided at the URL below, where users can find information related to data access: 10.57780/s3d-6c5ea43.

The whole reprocessed dataset has been validated by the MPC experts^4^. The main validation results are presented in the next sections. The products are generated at different latency times: Near Real Time (NRT, <3 hours latency), Short Time Critical (STC, <2 days latency), Non Time Critical (NTC, <1 month latency). The differences between NRT, STC and NTC products are in the input auxiliary data, for example the estimation of the satellite orbit. The data with the highest quality are produced in NTC latency.

For each Thematic Processing chain, two NetCDF data files are delivered to the users: a “standard” product and an “enhanced” product. The standard product essentially includes the main geophysical parameters derived from altimetry waveforms, along with the main geophysical and instrumental corrections applied to the altimeter range. The enhanced product includes the same variables as the standard one, and additional parameters dedicated to expert users, such as: the altimetry waveforms, all geophysical and instrumental corrections, the MWR brightness temperatures, parameters of the delay-Doppler stack. The detailed content of the Sentinel-3 STM Hydro-Cryo Thematic Products is described in the Product Data Format Specification (PDFS) document, available in SentiWiki website (“S3IPF PDS 003.2” document): https://sentiwiki.copernicus.eu/web/document-library#DocumentLibrary-SRAL.

The main product data content is displayed in Table 1, reporting the variables described in section ‘Surface topography retrieval’.

**Table 1:** Main parameters recorded in the Sentinel-3 Thematic Products.

	Hydrology Thematic Product	Sea Ice Thematic Product	Land Ice Thematic Product
**Main topography variable**	***elevation_ocog_20_ku**** OCOG/ICE-1 retracker, Bamber et al*.^15^	***freeboard_20_ku**** radar freeboard processing chain from Tilling et al*.^23^	***elevation_ocog_20_ku**** OCOG/ICE-1 retracker, Bamber et al*.^15^
**Measurement coordinates**	***lon_20_ku***/***lat_20_ku**** nadir coordinates*		***lon_cor_20_ku***/***lat_cor_20_ku**** POCA coordinates for Antarctica and Greenland measurements, from linear slope relocation, Envisat Handbook*^22^
**Backscatter coefficient**	***sig0_ocog_20_ku**** OCOG/ICE-1 retracker, Bamber et al*.^15^		
**Other useful topography information**	***range_water_20_ku*** altimeter range from SAMOSA retracker^16^	***sea_ice_ssha_20_ku*** Sea Surface Height Anomaly ***surf_type_class_20_ku*** Sea Ice classification: “open ocean/sea ice/lead/unclassified”	/

## Technical Validation

### Evaluation of Hydrology Thematic Products over lakes

The Hydrology Thematic Product was evaluated over a subset of worldwide lakes to access its quality. In this analysis, only lakes with an area larger than 20 km^2^ were considered, to ensure a reasonable amount of altimetry measurements per lake. This amounts to more than 4,100 lakes where a water level timeseries was reconstructed for each Sentinel-3A and Sentinel-3B track, via the R-package ‘tsHydro’^32^, resulting in approximately 7,300 timeseries. The timeseries model also estimates the standard deviation of the observations, enabling quantification of the noise level/stability of the Sentinel-3 surface water level. One observation standard deviation is estimated per timeseries, based on the dispersion of all 20 Hz water level measurements selected to build it (details regarding the processing are provided in the supplementary materials S4).

**Fig. 3:**
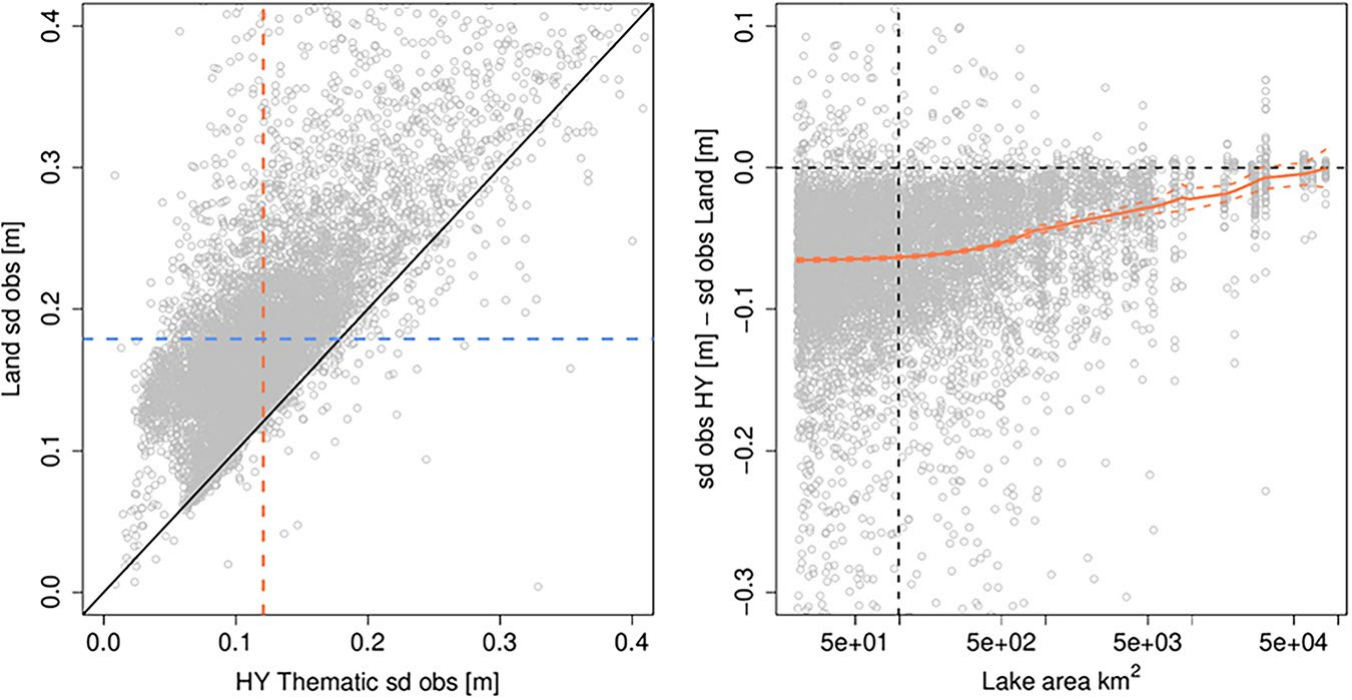
(Left) Estimated observation standard deviation, Hydrology Thematic (HY) vs Land Products. The dotted blue and orange lines represent the median of the estimated observation standard deviation of the Hydrology Thematic and Land Products, respectively. (Right) Difference in estimated observation standard deviation as a function of lake area. The vertical dotted black line indicated the median of the lake area. The orange line is a locally weighted regression fit.

In Fig. 3 (left), the observation standard deviations for each lake estimated from the Hydrology Thematic Product are plotted against the ones estimated from the former Land Product. The orange and blue dashed lines represent the median observation standard deviation of the new Hydrology Thematic and former Land Products, respectively.

In Fig. 3 (right), the difference in the estimated observation standard deviation between the Land and the Hydrology Thematic Products is displayed as a function of the lake area. From the point cloud and the locally weighted regression (LOWESS) shown with a solid orange line, an overall larger improvement is observed for lakes with an area below approximately 500 km^2^. On the contrary, the two products have a similar observation standard deviation for the largest lakes. The uncertainty (2σ) of the LOWESS fit is derived from bootstrap using 1000 replicas. In summary, we found that the new Hydrology Thematic Product provides more stable observations. More specifically, the median of the estimated observation standard deviation has decreased from 17 cm to 12 cm.

A gauge comparison was conducted to assess the capability of Sentinel-3 to monitor lake water level variations over time. Here, we used gauge data of water level with daily resolution from American (US) (USGS Water Data for the Nation, accessed January 10, 2024, at URL http://waterdata.usgs.gov/nwis/) and Canadian (Extracted from Environment and Climate Change Canada’s HYDAT.mdb, released on 2023-11-20) networks. R-scripts, to acquire the gauge data used in this evaluation, are provided as supplementary material. The Sentinel-3 water level timeseries remain computed as described in supplementary materials, section S4. The evaluation, with respect to the gauge level, was assessed via the statistical measures: Median Absolute Deviation (MAD), Root Mean Square Error (RMSE), Pearson correlation, and percentage of valid observations (i.e. observations within 0.5 m of the gauge level at the time of the altimetry measurements). In total, 629 altimetry-gauge pairs distributed over 185 lakes were formed. Hence, some lakes are represented by more pairs, as several gauges or satellite tracks are present at the same lake. To avoid replication, a water level timeseries based on a given track is only compared to the closest gauge. A bias, computed as the median difference between the gauge and altimetry-based water level reconstructed via the timeseries, was removed to account for differences in the vertical references. The medians of the statistical measures for all evaluations are reported in Table 2, for both Land and Hydrology Thematic Products. To quantify the performance of Sentinel-3 under ice-free conditions, the statistical measures were also calculated for the summer data only (from the 1^st^ of June to the 1^st^ of November), and reported in the parentheses in Table 2.

**Table 2:** Statistics summarising the agreement between Sentinel-3 and gauge data over US and Canadian lakes (over lakes with area higher than 20 km^2^). For each altimetry-gauge identified pair, a water level timeseries is retrieved from Sentinel-3 and gauge measurements, and statistical indicators (MAD, RMSE, Pearson correlation and Valid points) are used to analyse the difference between both timeseries. The median value of the statistics is reported in the table. The values in parentheses correspond to statistics computed under ice-free conditions, during summer period.

	# Pair	MAD [m]	RMSE [m]	Correlation	% Valid
**BC-005 Hydrology Thematic Products**	629 (599)	0.07 (0.05)	0.20 (0.09)	0.90 (0.95)	84 (87)
**former Land Products**	629 (599)	0.08 (0.06)	0.23 (0.10)	0.88 (0.95)	81 (83)

In summary, this highlights the capability of Sentinel-3 to monitor water level change over time, at least for lakes with an area above 20 km^2^. The summer-based statistics show an improved performance compared to year-round statistics, indicating a higher capability of Sentinel-3 to capture water level variations under ice-free conditions. We found that all reported evaluation measures are improved for the new Hydrology Thematic Product demonstrating its added value for inland water applications.

As can be deduced from Fig. 3 (right panel), the improvements obtained with the Thematic Products increase as lake size decreases. However, the relatively larger improvement in the estimated observation standard deviation (Fig. 3, left panel) at higher values is unrelated to the lake area. The noise level of the altimeter range was also assessed over relatively small lakes (between 1 km^2^ and 5 km^2^). This analysis is available in supplementary materials, section S5. Over these small-scale water bodies, it was found that the Hydrology Thematic Product provides a noise level reduction by about a factor of three, in comparison to the former Land Product. We underline that the delay-Doppler processing is now performed with a Hamming weighting window and a zero-padding technique in the Thematic IPFs (since BC-005 version). As mentioned in section ‘Delay-Doppler processing’, it is anticipated that these evolutions are mainly valuable in the situation of specular radar return in the range window analysis. Therefore, it is expected that the enhancements are primarily observed over the smallest water bodies, where the waveform peakiness parameter and the backscatter coefficient are theoretically higher compared to larger size lakes. The larger improvement for lakes with a higher estimated observation standard deviation might also be explained by the hamming window, since these lakes are less influenced by disturbances from the surroundings.

### Evaluation of Hydrology Thematic Products over rivers

In this analysis, the Sentinel-3 performance was evaluated over specific rivers with respect to *in-situ* measurements from the SCHAPI network. This network is composed of more than 1,500 georeferenced *in-situ* stations, monitoring various French rivers and canals. The *in-situ* stations provide WSH timeseries with centimeter-level accuracy and a sampling time of 15 min. WSH timeseries for each station is publicly available through the url: https://hydro.eaufrance.fr/rechercher/entites-hydrometriques.

In our comparative assessment, we first defined a subset of *in-situ* stations, by selecting those located within a 1.5 km distance to the Sentinel-3 theoretical ground tracks. This distance is a trade-off between comparison consistency, as the Sentinel-3 and *in-situ* measurements must sample the similar water area at each cycle, and sufficient *in-situ* stations kept for the analysis. For each of the 225 identified *in-situ* stations, we associated an altimetry “Virtual Station”, as commonly done for inland waters altimetry^33^. The location of the virtual station corresponds to the intersection point between the Sentinel-3 theoretical ground track and the monitored river. Using repeat track observations, Sentinel-3 WSH timeseries were computed at the virtual station location. For each orbit cycle, the Sentinel-3 measurement closest to the river centerline is selected within an area of 1 km around the virtual station (to consider the orbit excursion). The river centerline is determined using the “BD Topage” dataset: https://www.sandre.eaufrance.fr/atlas/srv/api/records/7fa4c224-fe38-4e2c-846d-dcc2fa7ef73e).

The Sentinel-3 and *in-situ* measurements must have a maximum time separation of four hours to be compared. The altimeter WSH is extracted from the Sentinel-3 product variable “*elevation_ocog_20_k*u”, available at 20 Hz rate. An example of Sentinel-3 WSH timeseries is presented in supplementary materials, section S6.

After determination of one Sentinel-3 WSH timeseries for each virtual station, we decided to remove from the timeseries the Sentinel-3 WSH measurements deviating by more than 1 m to the *in-situ* estimation. If more than 50% of Sentinel-3 measurements are discarded in the WSH timeseries, the virtual station was not considered in the performance evaluation. From the initial 225 virtual stations, 40 have been finally kept after this data editing. This drop can be explained by various factors. Specifically, inconsistencies with the *in-situ* observation can occur for measurements taken over steep river slopes, as the altimeter and the *in-situ* station are not observing the same water level. In addition, the altimeter range estimation is complicated in the case of several different watercourses sampled within the radar footprint^34^. Thus, the results obtained with this analysis are meant to be representative of the Sentinel-3 performance over isolated rivers (wide or narrow), with relatively low slope values.

**Fig. 4:**
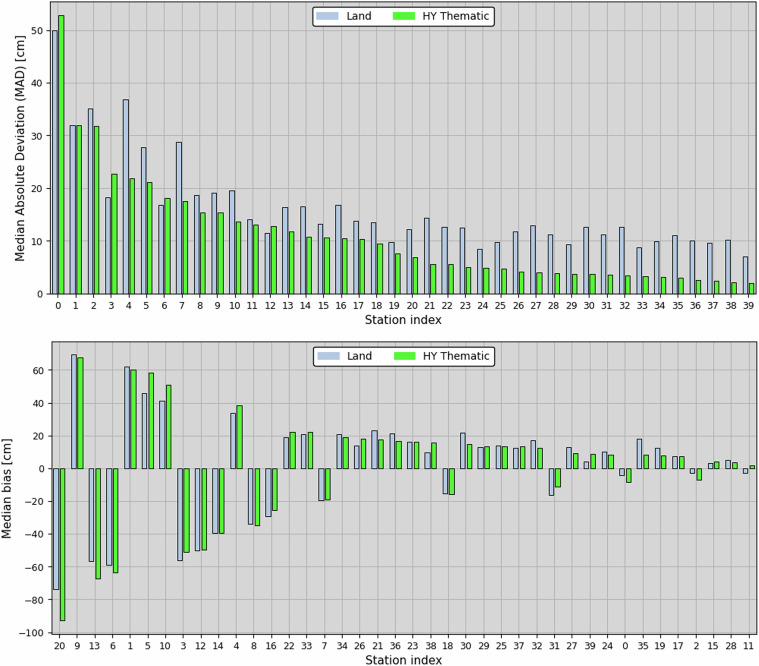
Median absolute deviation (top) and median bias (bottom) between water level timeseries from SHAPI *in-situ* measurements and Sentinel-3, over 40 *in-situ* stations. Sentinel-3 water level timeseries were constructed with the Hydrology Thematic (green bars) and former Land (blue bars) Products.

For each virtual station, we calculated the median bias and MAD between the WSH timeseries obtained with Sentinel-3 and SCHAPI measurements. The two statistics provide metrics related to the accuracy and precision of Sentinel-3, respectively. In Fig. 4, the MAD and median bias are displayed for each of the 40 *in-situ* stations included in the final dataset. The main improvement achieved with the Hydrology Thematic Product is in the measurement precision. The MAD estimated on the WSH timeseries is on average reduced by about 32.5%, in comparison to the former Land Products. This is consistent with results obtained over relatively small lakes, for which we demonstrated in supplementary materials S5 that the range noise level is significantly lowered. In terms of measurement accuracy, the bias relative to the *in-situ* stations remains on average comparable between Land and Thematic Products. The mean of the median bias differences between Hydrology Thematic and Land Products is estimated at +6 mm (calculated as Thematic - Land). As it can be noticed in Fig. 4, bottom panel, the Sentinel-3 accuracy exhibits a variability of the order of several decimetres, from one *in-situ* station to another. The variability of this bias, besides other factors (river slope, contaminants, etc.) already mentioned, is potentially linked to the OCOG/ICE-1 retracker, implemented in both Land and Hydrology Thematic Products. This retracking algorithm can be classified as an empirical “threshold retracker”. With this type of retracker, the altimeter range is derived by finding the first waveform sample crossing a power threshold. To our knowledge, this threshold remains to be adjusted for river measurements. Physical retracking algorithms will also be studied by the MPC experts, and envisaged for future PB, to further improve the accuracy of Sentinel-3 altimetry measurements over rivers.

### Evaluation of Sea Ice Thematic Products - availability of freeboard measurements

**Fig. 5:**
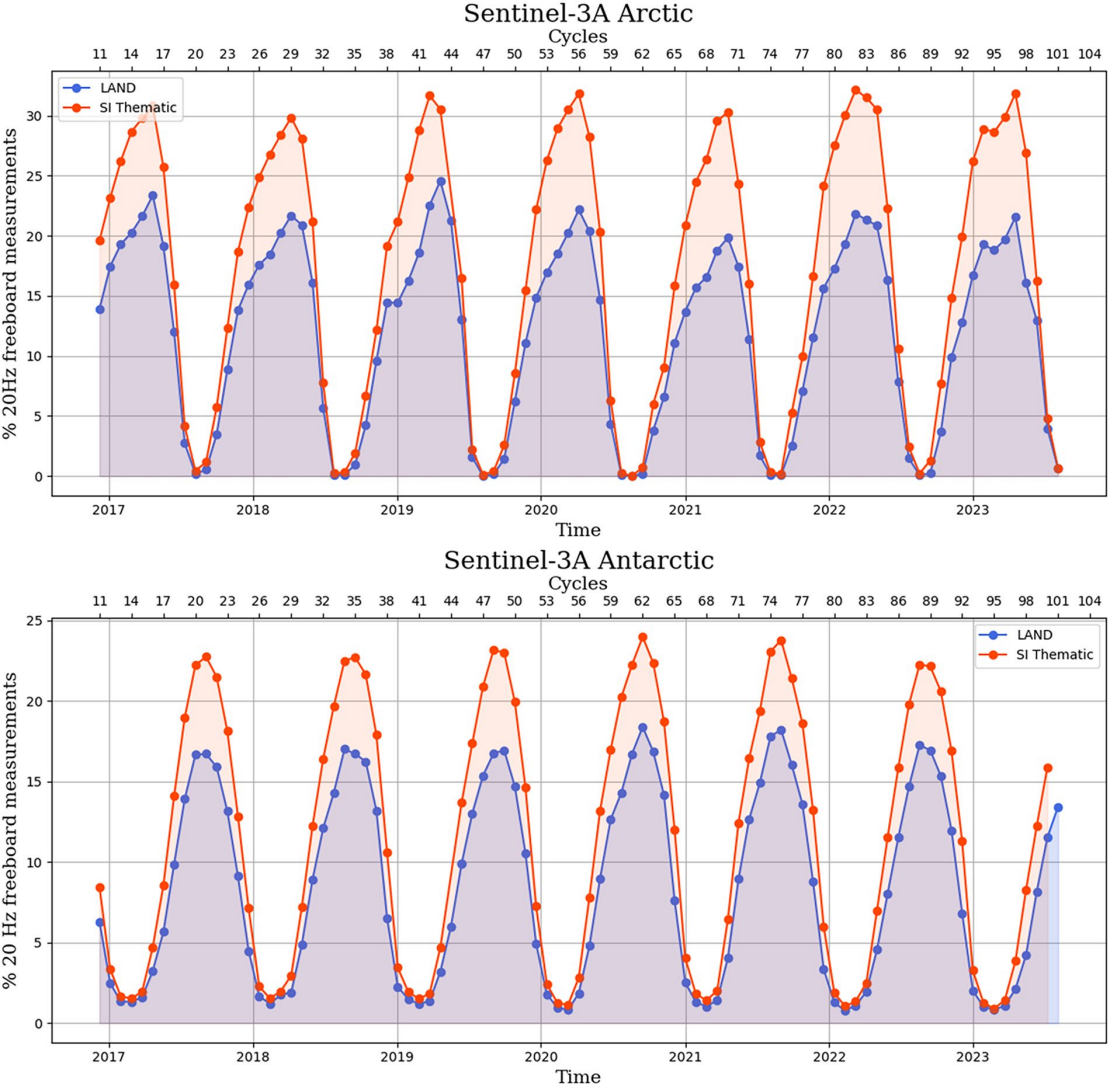
Percentage of valid radar freeboard points (valid = not at default value) over the total number of measurements in the static Sea Ice thematic mask, computed along-track for each Sentinel-3A cycle throughout the whole Sentinel-3A mission, for the Arctic (top panel) and the Antarctic (bottom panel).

In a first assessment, we controlled the Sea Ice Thematic Product dataset by quantifying the number of freeboard measurements successfully derived by the ground segment processing. In Fig. 5, the percentages of 20 Hz freeboard measurements available in the former Land and new Sea Ice Thematic products are displayed, over both the Arctic and the Antarctic sea ice, for the Sentinel-3A unit. These percentages are defined as the ratio between the number of 20 Hz freeboard records successfully derived by the processor, and the total number of 20 Hz records available within the static sea ice thematic mask (presented section ‘Overview of Sentinel-3 Hydro-Cryo Thematic Processors and Products’). For the Arctic sea ice (top panel), the number of freeboard records contained in the Sentinel-3 products is the highest at the end of the Arctic winter (March/April), when sea ice extent reaches its maximum. For the Antarctic sea ice, the maximum occurs towards the coldest month of the Antarctic winter, usually between August and September. As shown in Fig. 5, the percentage of 20 Hz freeboard records available in the Thematic Product has significantly increased, in comparison to Land Products. This gain is estimated approximately at 25% and 30%, over the Antarctic and Arctic sea ice, respectively.

These improvements are explained by different factors acting in the sea ice classification implemented in the Sentinel-3 ground segment processing. The goal of this classification is to identify the surface type associated with each 20 Hz altimetry record, between: open ocean, leads and sea ice. This is achieved with a combination of thresholds, applied to both the peakiness of the SAR mode waveform and the sea ice concentration read from auxiliary data^23^. An “unclassified” category also exists in the case of measurements detected as ambiguous. With the new Thematic processing, we noticed a reduction of the “unclassified” measurements, explaining the higher availability of freeboard records. An analysis of the sea ice classification outputs is provided in the supplementary materials, section S7. The decrease of unclassified records is most likely explained by the difference in the waveform shape between the two product versions. The modification of the waveform shape is itself induced by the upgraded delay-Doppler processing, performed with Hamming weighted window and zero-padding, as explained section 2.2, and as illustrated in supplementary materials, section S2. Moreover, additional freeboard measurements are also available close to the coastlines, thanks to the update made on the sea ice concentration model^35^. It is finally worth noting that similar conclusions are reached with the Sentinel-3B unit, this is documented in the BC-005 reprocessing validation report written by the Sentinel-3 MPC^4^.

### Evaluation of Sea Ice Thematic Products – reconciliation with CryoSat-2

Launched in 2010 by ESA, CryoSat-2 is a radar altimetry mission primarily dedicated to the observation of the cryosphere. The on-board altimeter operates in SAR mode over the sea ice areas, with an instrumental configuration close to Sentinel-3 SRAL^36^. Cryosat-2 also operates in SARIn (Synthetic Aperture Radar Interferometry) mode in dedicated areas, mainly close to the shoreline in the polar zones. Thanks to its nearly polar (88° inclination) and geodetic orbit, CryoSat-2 is a reference altimetry mission for the sea ice community. In the ESA ground segment processing (ICE Baseline), as for the Sentinel-3 Sea Ice Thematic IPF, the delay-Doppler is performed with Hamming weighting window and zero-padding techniques with a factor of two^37^. In this assessment, we performed a comparison of the radar freeboard available in the Sentinel-3 Sea-Ice Thematic, Sentinel-3 Land and Cryosat-2 ICE Products(Baseline-E). The CryoSat-2 Products were downloaded from the ESA ftp: “ftp://science-pds.cryosat.esa.int”, data references: “SIR_SAR_L2” and “SIR_SIN_L2” (last access December 2024). The measurements analysed were acquired during the same period, from 1 January to 1 March, 2020, for the Arctic, and from 1 July to 1 September, 2019, for the Antarctic.

**Fig. 6:**
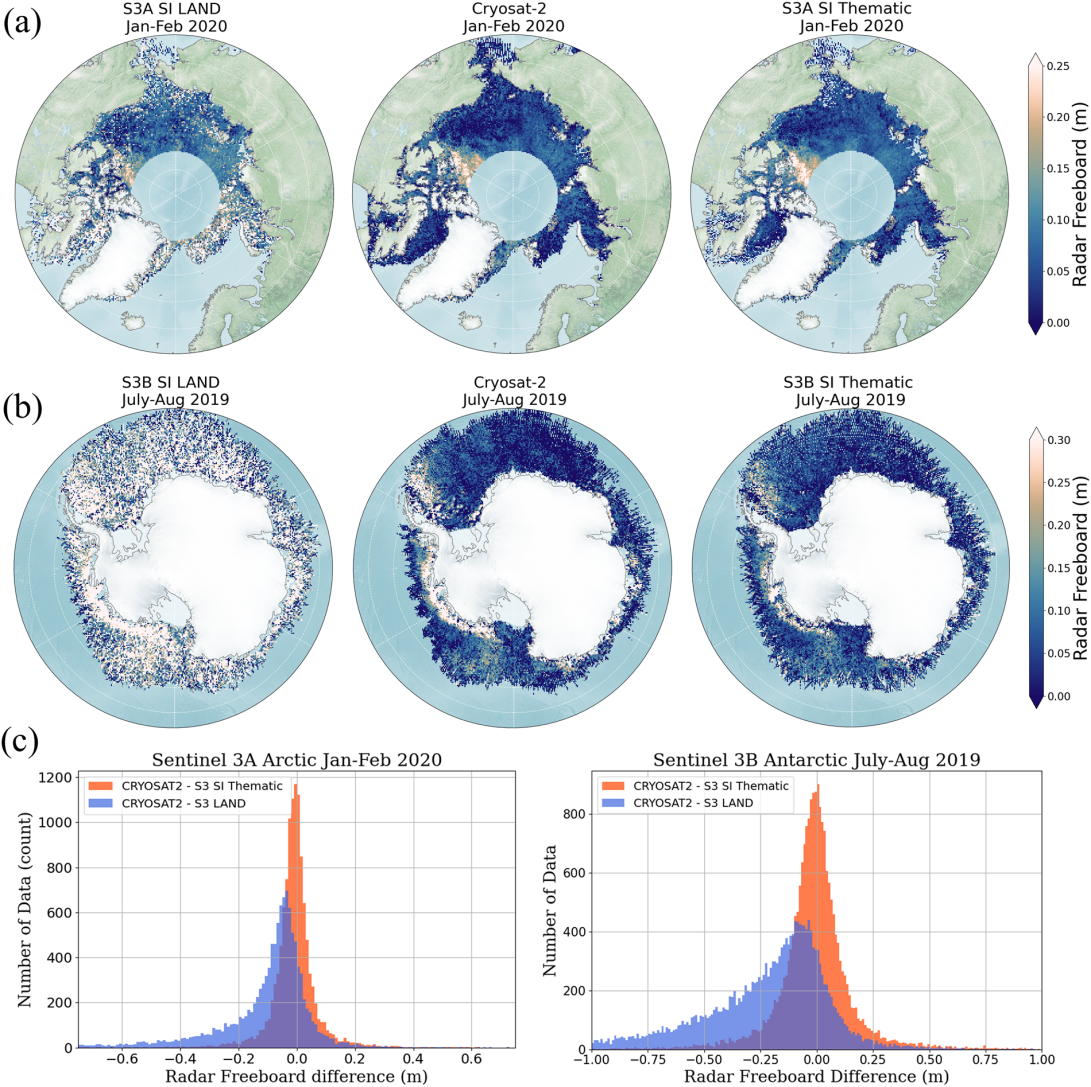
Top and middle rows are the gridded maps (25 km × 25 km) of the radar freeboard for (**a**) the Arctic winter for Sentinel-3A (January-February 2019) and (**b**) the Antarctic winter for Sentinel-3B (July-August 2019). From left to right: Sentinel-3 Land Product, Cryosat-2 Baseline E (limited to +/− 81.5° in latitude and computed over the same period) and Sentinel-3 Sea Ice Thematic Product. Last row (**c**) displays the corresponding [CryoSat-2-Sentinel-3] difference histograms for the Arctic (left) and the Antarctic (right), computed over the grids.

In Fig. 6a,b, the 20 Hz freeboard measurements are mapped using a 25 km stereographic grid. To make a fair and consistent comparison , latitude has been limited to ± 81.5° for Cryosat-2. The freeboard differences between Cryosat-2 and Sentinel-3, computed over the gridded points, are displayed as histograms in Fig. 6c. Table 3 summarises the key metrics: for each hemisphere, the mean, median and standard deviation values are provided for the freeboard computed using Sentinel-3 Land, Sentinel-3 thematic and Cryosat-2, along with the statistical differences between the three Products.

**Table 3:** Summary of the radar freeboard statistics obtained for the Sentinel-3 Land Products, the Sentinel-3 Sea-Ice Thematic Products, and the Cryosat-2 ICE Products, computed over two winter months for the Arctic (S3A January/February 2020) and the Antarctic winter (S3B July/August 2019) using 25 km × 25 km grids.

Radar Freeboard	Arctic [January 2019]					Antarctic [July/Aug 2019]				
	S3 LAND	CS2	S3 Thematic	CS2 - S3 Land	CS2 - S3 Thematic	S3 LAND	CS2	S3 Thematic	CS2 - S3 Land	CS2 - S3 Thematic
Mean (cm)	12.2	5.8	6.1	**−13.6**	**−1.0**	32.0	4.8	6.5	**−28.2**	**−1.2**
Median (cm)	19.5	5.1	5.8	**−6.7**	**−0.8**	20.5	4.7	6.1	**−17.6**	**−0.9**
STD (cm)	33.8	23.1	21.6	**31.5**	**19.0**	42.6	20.4	21.0	**43.2**	**20.4**

These metrics are complementary to Fig. 6.

The gridded maps reveal an apparent performance improvement of the Sentinel-3 Sea Ice Thematic Product compared with the Land Product. Firstly, in terms of variability for the Arctic, the standard deviation obtained with the Thematic Product is significantly reduced (22 cm) compared to the Land products (34 cm) and is comparable to Cryosat-2 (23 cm). For the Antarctic sea ice, the improvement is more significant, as the histogram standard deviation decreases from 43 cm to 21 cm, and is thus comparable to the 20 cm obtained with CryoSat-2. Looking at the spatial variations, the radar freeboard estimated with Sentinel-3 is now in closer agreement with CryoSat-2. For instance, similar patterns can be observed in the maps, such as the typical multi-year sea-ice patch towards Queen Elizabeth islands^38^. This is confirmed with the histograms of the grid differences between CryoSat-2 and Sentinel-3 (Fig. 6c). With the Sea Ice Thematic Product, the histogram computed over the Arctic shows a Gaussian shape, less spread and almost centered around zero. The Cryosat-2/Sentinel-3 median bias (computed using the grid differences) is indeed significantly reduced, from -6.7 cm with the Land products to -0.8 cm with the Sea Ice Thematic Product.

The conclusions are similar over the Antarctic sea ice, as the median bias is also significantly reduced, from -17.6 cm with the Land products with respect to -0.9 cm with the Sea Ice Thematic Product. The relatively small remaining differences between Sentinel-3 and CryoSat-2 can be explained by different choices made in the radar freeboard calculation. While the same retracking algorithms are implemented in the two ground segments, differences exist at other processing stages. For instance, in the CryoSat-2 ground segment, the sea ice classification is made using information from the delay-Doppler stack (in addition to the waveform peakiness and sea ice concentration parameters). The interpolation of the sea ice surface topography is also performed differently since CryoSat-2 ICE Baseline-E.

In this assessment, the analyses were conducted with the Sentinel-3A unit for the Arctic, and the Sentinel-3B unit for the Antarctic. It must be noted that similar results are observed between the two Sentinel-3 units on both hemispheres, as documented in the BC-005 reprocessing validation report, written by the Sentinel-3 MPC^4^. In conclusion, the former version of the Sentinel-3 products was not performing well enough to be exploited by sea ice users, and first assessments of the potential of Sentinel-3 for sea ice research required custom processing chains^39^. In contrast, the Thematic Product now provides radar freeboard measurements closely aligned to those available from CryoSat-2 ICE Baseline Products.

### Evaluation of Sea Ice Thematic Products – comparison against airborne measurements

**Fig. 7:**
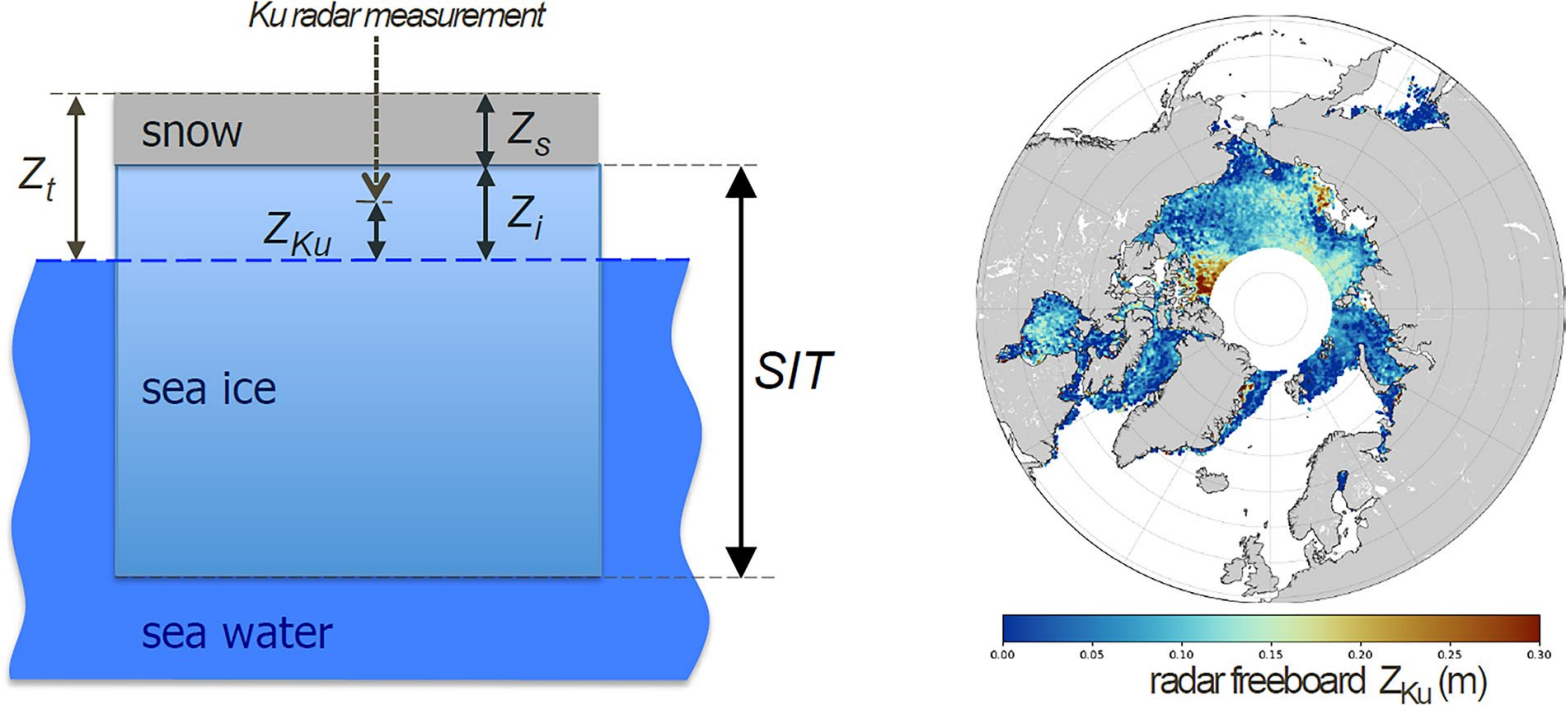
(left) Main sea ice floe dimensions. The “radar freeboard” (Z_ku_) is smaller than the “sea ice freeboard” (Z_i_), due to the radar wave penetration into the snow medium in Ku-band. (right) Gridded radar freeboard Z_Ku_ measured by Sentinel-3A in April 2018.

The Sentinel-3 radar freeboard provided in the Sea Ice Thematic Products (variable “*freeboard_20_ku*”) was compared with Operation Ice Bridge (OIB) airborne measurements from the National Aeronautics and Space Administration (NASA)^40^. OIB campaigns are carried out each spring in Western Arctic from 2009 to 2019. The aircraft was equipped with a laser altimeter and a snow radar, giving access to the sea ice freeboard “*Z*_*i*_*”* and the snow depth “*Z*_*s*_*”*, from which the total freeboard “*Z*_*t*_*”* can be computed: *Z*_*t*_ = *Z*_*i*_ + *Z*_*s*_, as illustrated in Fig. 7.

With the knowledge of the total freeboard and the snow depth, the Sea Ice Thickness (SIT) can be computed with the hydrostatic equilibrium equation:

1 $${airborne\; Sea\; Ice\; Thickness}\left({SIT}\right)=\frac{\rho w\,{Z}_{t}+(\rho s-\rho w){Z}_{s}}{(\rho w{\rm{\mbox{--}}}\rho i)}$$

where *ρ*_*w*_*, ρ*_*i*_ and *ρ*_*s*_ are the densities of the water, the ice and the snow, respectively. Typical mean values are used for these parameters: *ρ*_*w*_ = *1024 kg/m*^3^*, ρ*_*i*_ = *900 kg/m*^3^*, ρ*_*s*_ = *300 kg/m*^3^ (ref. ^41^).

Using the same snow depth “*Z*_*s*_*”*, taken from OIB measurements, the Sentinel-3 sea ice thickness can be computed using the radar freeboard estimated by the altimeter: “*Z*_*ku*_“, with the following hydrostatic equilibrium equation from Mallett *et al*.^41^:

2 $${satellite\; Sea\; Ice\; Thickness}\left({SIT}\right)=\frac{\rho w\,{Z}_{{Ku}}+(\rho w\,\frac{{c}_{v}}{{c}_{s}}-\rho w+\rho s){Z}_{s}}{(\rho w{\rm{\mbox{--}}}\rho i)}$$

where *c*_*v*_*/c*_*s*_ is the ratio of the speed of propagation of the radar in the vacuum and in the snow. From Ulaby *et al*.^42^, this ratio can be expressed as *c*_*v*_*/c*_*s*_ = *(1* + *0.00051 ρ*_*s*_*)*^*1.5*^.

**Fig. 8:**
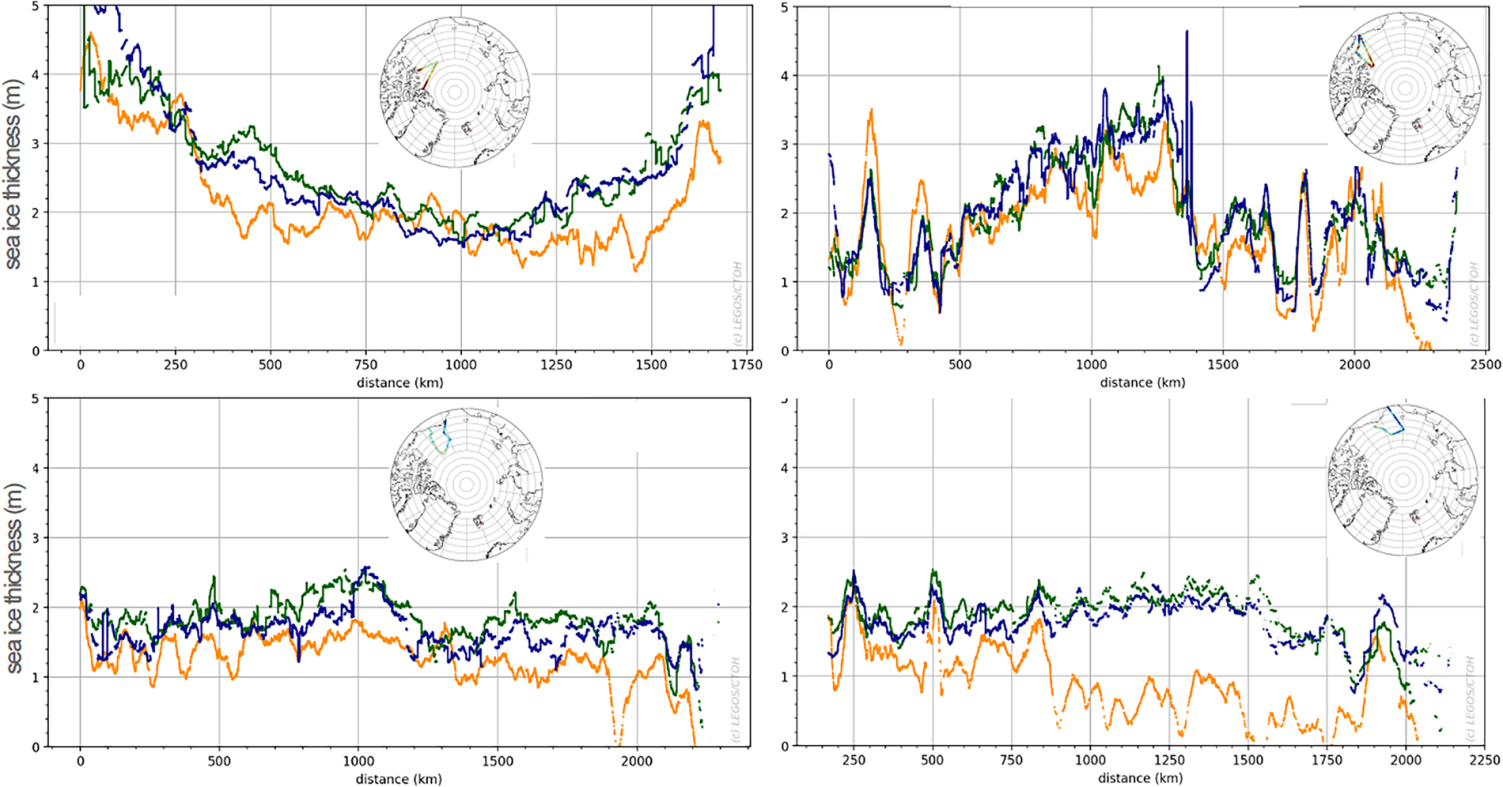
Comparison of Sea Ice Thickness along Operation IceBridge (OIB) measurements (in orange) and interpolated from monthly radar freeboard maps (Sentinel-3A in green; and CryoSat-2 in blue), for the OIB acquisitions from 2017/04/19, 2018/04/14, 2018/04/07 and 2018/04/08. The OIB flight trajectories are displayed in the background maps, with OIB Sea Ice Thickness displayed with a scale colour from 0 to 3 m.

As the airborne and the satellite flights are not co-located, the Sentinel-3 radar freeboard available in the Sea Ice Thematic Products has been gridded into monthly maps of 12.5 km resolution, using a smoothing radius of 25 km, as displayed in Fig. 7, right panel. The same methodology has been also applied to CryoSat-2 measurements, with radar freeboard extracted from the ICE Baseline-E Products disseminated by ESA (the data access information is provided in the previous analysis). The Sentinel-3 and CryoSat-2 radar freeboards are finally bi-linearly interpolated in the monthly maps, along the airborne flights of the corresponding month. The interpolated freeboards are converted to SIT following Eq. (2), with the airborne snow depth measurements “*Z*_*s*_” retrieved along the OIB flights. Figure 8 shows comparisons of the along-track SIT estimated from Sentinel-3, CryoSat-2 and OIB for the four available flights below 81.5°N.

Given the strong dynamics of the sea ice (a few kilometres per day), and possible climatic contingencies over the course of a month (snowfall, melting, wind), the differences between instantaneous airborne measurements and monthly spatial measurements can be consequent, in particular in front of Bering Strait (bottom right panel in Fig. 8). However, for the three other cases, the SITs derived from airborne and space measurements show similar level and follow the same major variations. This result is promising, highlighting the capability of satellite measurements to retrieve a consistent radar freeboard at few tens of kilometre scale.

### Evaluation of Land Ice Thematic Products – data coverage improvement

The major change introduced in the Land Ice Thematic IPF is the delay-Doppler processing with extended window. This technique was shown to be valuable over the ice sheet margins, to recover backscattered energy in the delay-Doppler stack^14^. The impact of this evolution was assessed by examining the quality of the SAR mode waveforms over the Antarctic and Greenland ice sheets. The waveform quality was determined through two criteria. Firstly, a backscatter coefficient was calculated for each of the along-track measurements. A -12 dB threshold was set to flag the low Signal-to-Noise Ratio (SNR) measurements. Additional information related to the Sigma-0 calculation, and this threshold definition, is available in supplementary materials, section S8. Secondly, the presence of an energy peak in the radar waveform was controlled using the Leading Edge Detection (LED) algorithm. This algorithm is inspired from the Canny edge detector^43^, to detect waveform energy peak(s) in radar altimetry waveforms, and is described in Aublanc *et al*.^44^. In the absence of a waveform energy peak, or in case of low SNR, the measurement is flagged as “invalid”.

**Fig. 9:**
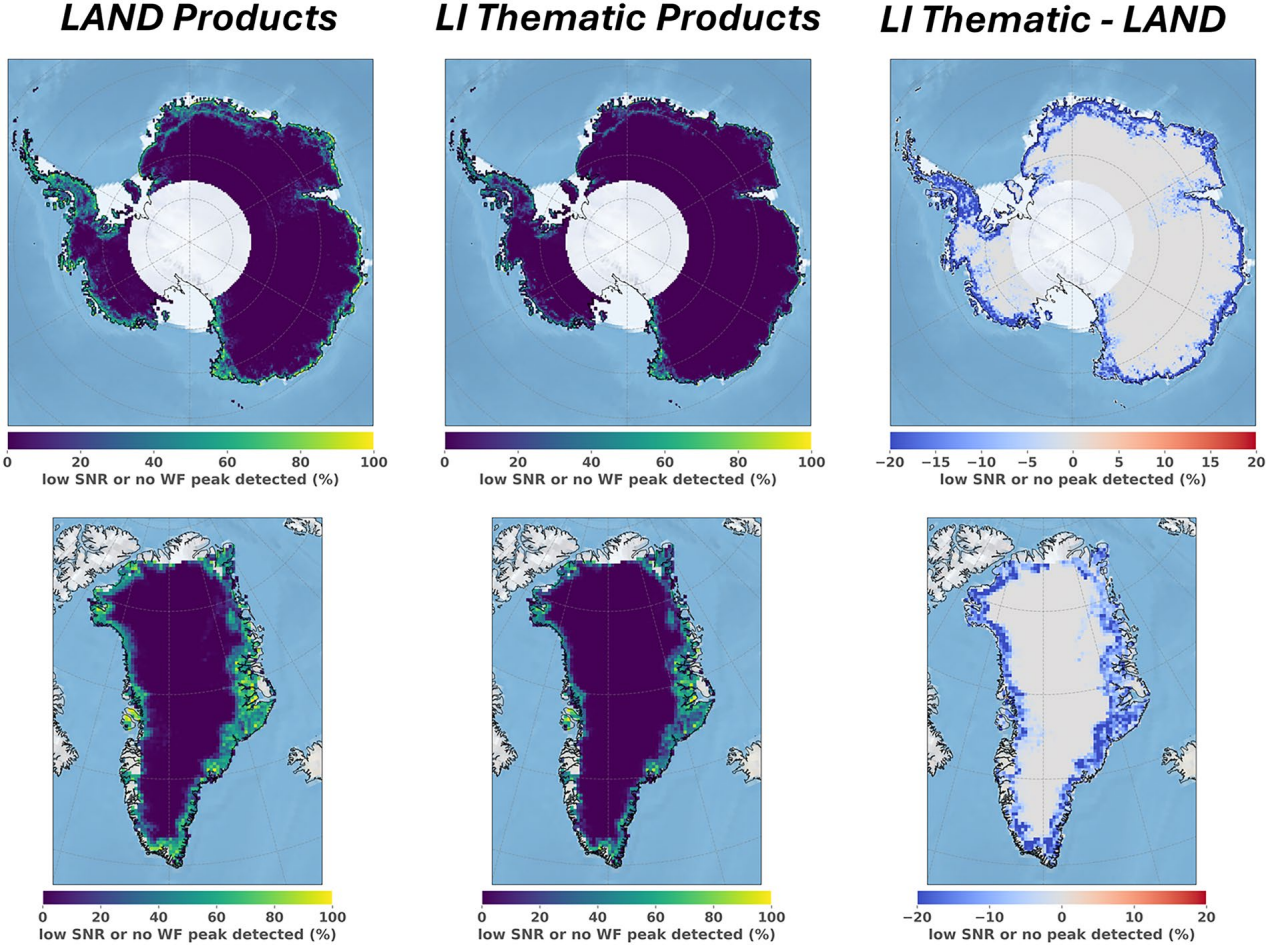
Average ratio of invalid Sentinel-3 SAR mode measurements, mapped over the Antarctic (top) and Greenland (bottom) ice sheets, using a 25 km polar stereographic grid. A measurement is considered “invalid” in case of low SNR, or if no clear energy peak is detected in the radar waveform. (left) Average ratio for the former Land Product. (center) Average ratio for the BC-005 Land Ice Thematic Product. (right) Difference between Land Ice Thematic and Land Products (computed as “Thematic – Land”).

The measurements were spatially selected over the polar ice sheets (ice shelves not included) using the surface flag from the BedMachine dataset (version n°3 over Antarctica; version n°5 over Greenland; Morlighem *et al*.)^29^. Eleven orbit cycles of Sentinel-3A and Sentinel-3B were analysed, corresponding to 297 days of acquisitions, from January to November 2022. The population of 20 Hz measurements selected for both Sentinel-3A and Sentinel-3B units is about 37,700 million over the Antarctic ice sheet, and 5,800 million over the Greenland ice sheet. In Fig. 9, the mean ratio of measurements considered “invalid” is mapped using a 25 km polar stereographic grid. The grid difference between Land and Land Ice Thematic Products is shown in the figures to the right.

With the former Land Product, the ratios of invalid measurements are, on average, estimated at 5.4% and 8.9%, over the Antarctic and Greenland ice sheets, respectively. With the Land Ice Thematic Product, the same ratios are 2.3% and 4.8%. Thus, approximately 3.1% and 4.1% of additional useful measurements are recovered with the Land Ice Thematic Product, over the Antarctic and Greenland ice sheets, respectively. As expected, the improvement is substantial over the ice sheet margins. For instance, where the surface slope is greater than 0.5°, the ratio of invalid measurements decreases from 32.5% (Land Product) to 15.3% (Land Ice Thematic Product) over the Antarctic ice sheet. The same ratios are 29.2% (Land Product) and 15.2% (Land Ice Thematic Product) over the Greenland ice sheet.

### Evaluation of Land Ice Thematic Products – accuracy and precision compared to ICESat-2 ATL06

The objective of this assessment is to quantify the accuracy and precision of the Sentinel-3 Land Ice Thematic Products over the Antarctic and Greenland ice sheets. For this purpose, the along-track elevations provided in the Land Ice Thematic Product (variable: “*elevation_ocog_20_ku*”) are compared to ICESat-2 ATL06 estimations^45^. The NASA ICESat‐2 mission launched in September 2018 carries a single instrument on board: the Advanced Topographic Laser Altimeter System (ATLAS), a photon‐counting laser altimeter using 532 nm wavelength laser pulses. The ATL06 product contains land ice elevations, posted at 40 m along the six ground track beams. ICESat-2 ATL06 elevations over the Antarctic ice sheet interior have a reported accuracy and precision respectively of about 3 cm and 7 cm^46^. Therefore, ICESat-2 ATL06 is considered a reliable topography reference, used in this context to evaluate the performance of the Sentinel-3 mission over ice sheets.

**Fig. 10:**
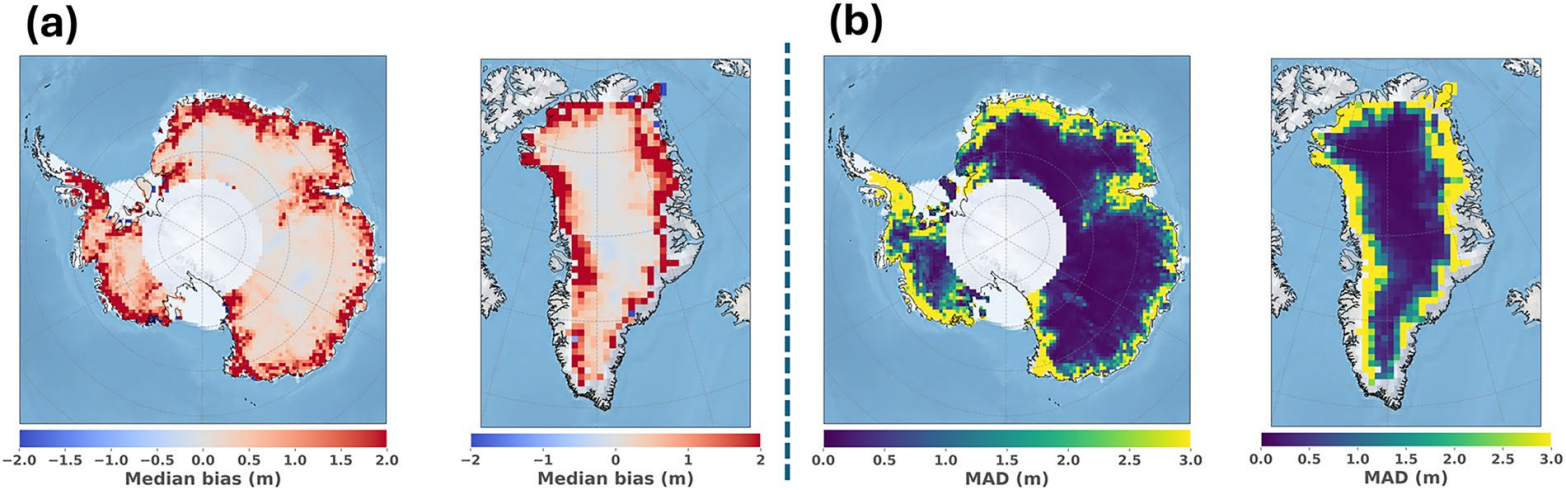
(**a**) Gridded median elevation bias between Sentinel-3 and ICESat-2 ATL06 colocated measurements over the Antarctic and Greenland ice sheets. Elevations are computed as Sentinel-3 – ICESat-2. (**b**) Gridded Median Absolute Deviation (MAD) bias between Sentinel-3 and ICESat-2 ATL06 colocated measurements over the Antarctic and Greenland ice sheets. Grid resolution is 50 km.

**Fig. 11:**
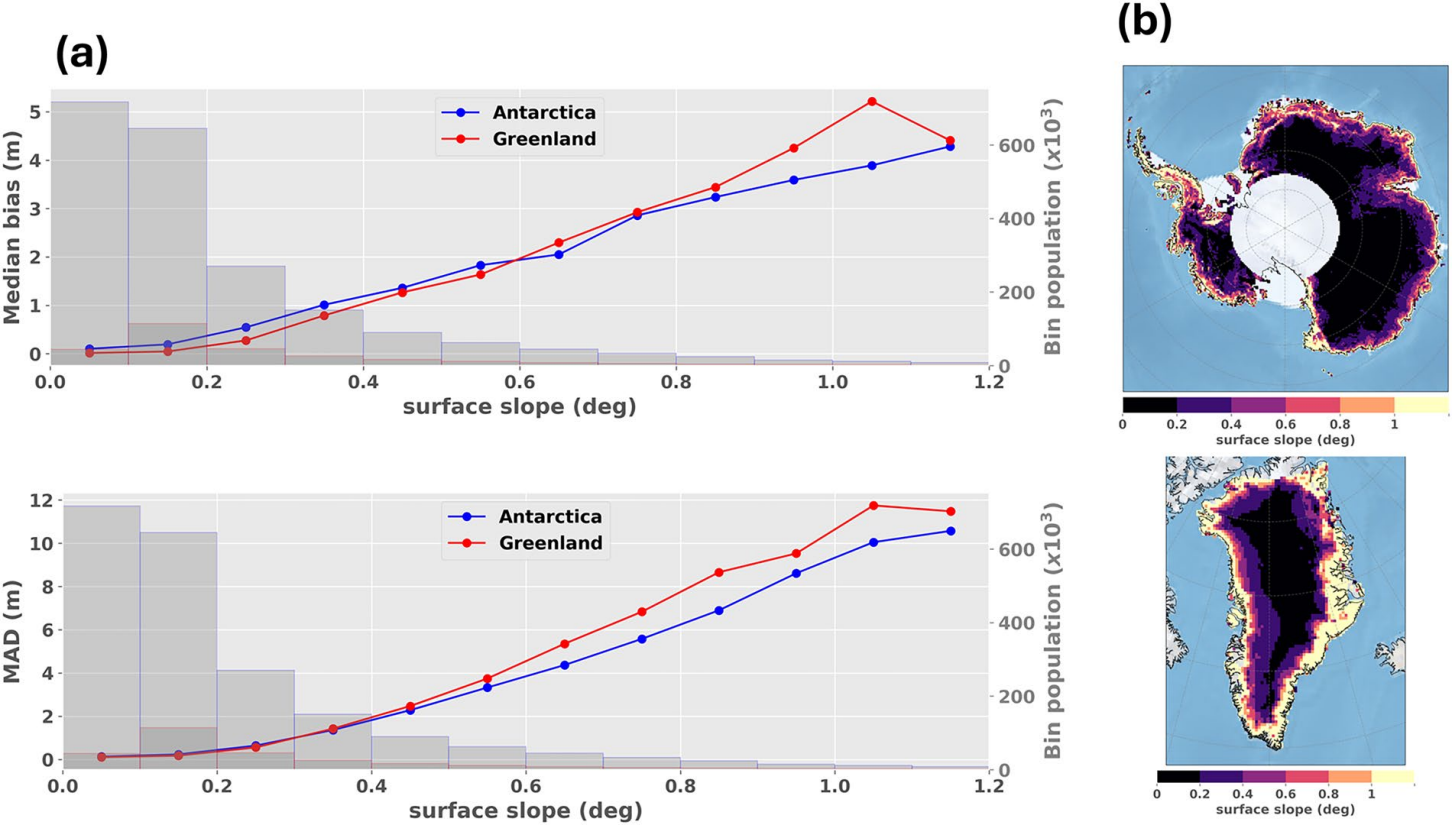
(**a**) Median bias (top) and Median Absolute Deviation (bottom) between Sentinel-3 and ICESat-2 ATL06 colocated elevations, represented as a function of surface slope. The statistics are computed over the Antarctic (blue curve) and Greenland (red curve) ice sheets. Elevation differences are computed as Sentinel-3 – ICESat-2. Grey bars display the number of colocated measurements for each slope interval. (**b**) Surface slope at the nadir of the measurements.

The near-coincident altimeter measurements from both data products were compared, using a search radius of 25 meters, along with a maximum time span between acquisitions of 46 days (corresponding to the ICESat-2 orbit cycle duration). The same subset of the Sentinel-3 Land Ice Thematic Product as presented in the previous section was assessed. The ICESat-2 bad quality data were discarded using the binary product flag “*atl06_quality_summary*”. Sentinel-3 and ICESat-2 nearly colocated measurements deviating by more than 100 m in elevation were considered as “outliers” and removed from the statistics. In Fig. 10, the median bias and MAD of the elevation differences between nearly colocated Sentinel-3 and ICESat-2 measurements are mapped using a 50 km polar stereographic grid. The same statistics are displayed in Fig. 11, represented as a function of the surface slope derived from the Reference Elevation Model of Antarctica (REMA)^47^, and ArcticDEM^48^. The surface slope is computed over a horizontal distance of ±7.5 km around nadir location (corresponding approximately to the -3 dB antenna beamwidth).

In terms of elevation accuracy, the results show that Sentinel-3 is relatively close to ICESat-2 ATL06 over the ice sheet interiors. The median elevation bias between Sentinel-3 Land Ice Thematic Product and ICESat-2 ATL06 is +0.13 m and +0.03 m where the surface slope is below 0.2°, over the Antarctic and Greenland ice sheets, respectively. However, Sentinel-3 overestimates the surface elevation over the ice sheet margins in comparison to ICESat-2 ATL06. For instance, where surface slope is between 0.5° and 1°, the median bias to ICESat-2 ATL06 increases to +2.27 m and +2.32 m, over the Antarctic and Greenland ice sheets, respectively. In terms of elevation precision, the MAD between Sentinel-3 Land Ice Thematic Product and ICESat-2 ATL06 is 0.17 m and 0.15 m where the surface slope is below 0.2°, over the Antarctic and Greenland ice sheets, respectively. The values increase to 4.67 m and 5.56 m where surface slope is between 0.5° and 1°.

Overall, the performance between the former Land and new Land Ice Thematic Products was found very comparable when compared to ICESat-2 ATL06. Nevertheless, an improvement in precision over the ice sheet margins is observed with the Thematic Product. It is most likely induced by the increase of the measurement quality over these areas, as reported in the previous section. A comparative analysis between Land and Land Ice Thematic Products is available in supplementary materials, section S9. In addition, the different assessments performed by the MPC confirm that both Sentinel-3A and Sentinel-3B units are performing equivalently over ice sheets, as also reported in McMillan *et al*.^49^. Over lake Vostok, the elevation bias between the two units is at the cm level, as estimated at track crossover locations^50^. As a main perspective, it must be noted that Sentinel-3 accuracy and precision can be significantly improved over the ice sheet margins, by using recent innovative algorithms for the echo relocation at POCA^30,51^. In fact, the relocation method implemented in the ground segment processing assumes a constant slope in the radar footprint to determine the POCA location. With this hypothesis, by nature, the relocation becomes less accurate over the ice margins, where topography is more irregular compared to the ice sheet interiors (at hm-km scale roughness).

## Supplementary information

Supplementary information

## Data Availability

The source code implemented in the Sentinel-3 Thematic IPFs is not openly accessible, but it is documented in Algorithm Theoretical Baseline Documentation (ATBD), with document identifiers “*S3MPC.ATBD.HY*”, “*S3MPC.ATBD.LI*” and “*S3MPC.ATBD.SI*”. https://sentiwiki.copernicus.eu/web/document-library#DocumentLibrary-AlgorithmTechnicalBaselineDocumentationLibrary-S3-SRAL-ATBD. Information about data processing and PB evolutions is available on SentiWiki website: https://sentiwiki.copernicus.eu/web/altimetry-processing.
