# Supplementary material for: Sentinel-3 Altimetry Thematic Products for Hydrology, Sea Ice and Land Ice
Source: Sci Data. 2025 Apr 29;12:714. doi: 10.1038/s41597-025-04956-3 (PMC12041192; doi:10.1038/s41597-025-04956-3)
Supplement: Supplementary file 1 — Supplementary information [file 41597_2025_4956_MOESM1_ESM.pdf]

Sentinel-3 Altimetry Thematic Products for Inland Waters, Sea Ice and Land Ice

Supplementary materials

Supplementary S1: Geophysical corrections used in the surface topography computation

(section 2.1: Overview of Sentinel-3 Hydro-Cryo Thematic Processors and Processing)

a) Hydrology and Land Ice Thematic Products

Where the altimeter is over the continental surfaces, the geophysical corrections listed below are applied to the product variable "***elevation_ocog_20_ku***".

It must be noted that the surface type information is provided through the product variable "***surface_type_20_ku***". The following corrections are applied over "*enclosed_sea_or_lake*" (flag value set to "1"), "*continental_ice*" (flag value set to "2") and "*land*" (flag value set to "3").

- **Atmospheric effects**

The time delay induced by the **dry and wet troposphere** is calculated at the nadir surface elevation, using vertical integration of 3D meteorological parameters, extracted from ECMWF Integrated Forecasting System (IFS) meteorological data. The corrections are available in the product variables "*mod_dry_tropo_cor_meas_altitude_01"* and "*mod_wet_tropo_cor_meas_altitude_01".*

The time delay induced by the **ionosphere** is extracted from an auxiliary data model: the JPL Global Ionosphere Maps (GIM) model (Iijima et al., 1999). The correction is available in the product variable "*iono_cor_gim_01_ku".*

- **Tidal effects**

The **Solid Earth Tide correction** removes the deformation of the Earth due to tidal forces from the Sun and Moon acting on the Earth's body. Typically, this correction ranges from -30 to +30 cm. The correction is computed using a static auxiliary file, derived from the Cartwright tide model (Cartwright and Edden, 1973). The correction is available in the product variable "*solid_earth_tide_01*".

The **Geocentric Polar Tide correction** accounts for the variations of the Earth's crust deformations caused by small perturbations in the Earth's rotation axis. It is computed as described in Desai (2015). The correction is available in the product variable "*pole_tide_01*".

The **Ocean Loading Tide correction** accounts for the deformation of the Earth's crust in response to the ocean tides. It is computed from FES 2014 model (Lyard et al., 2006). The correction is available in the product variable "*load_tide_sol2_01*".

b) Sea Ice Thematic Products

For Sea Ice Thematic Products, the same corrections as listed before are used to retrieve the radar freeboard. In addition, the topography is also corrected from:

- **Oceanic tide**

The "**Oceanic Tide**" correction accounts for gravitational effects acting on the ocean surface. The correction is provided from FES 2014 model (Lyard et al., 2006), and available in the field "*ocean_tide_sol2_01*" (this variable includes the sum of both "oceanic tide" and "ocean loading tide" corrections).

- **Inverse Barometer correction**

The Inverse Barometer (IB) correction accounts for variations in sea surface height due to atmospheric pressure variations (atmospheric loading) with wind effects ignored. The correction is calculated using the surface atmospheric pressure derived from ECMWF IFS meteorological data, and available in the parameter "*inv_bar_cor_01*".

As mentioned in the main text, prior to radar freeboard computation, the sea ice surface topography is referenced to a Mean Sea Surface (MSS), and is therefore defined as a "surface height anomaly" (product variable "*sea_ice_ssha_20_ku*"). The DTU21 model (Andersen et al., 2023) is used as the reference MSS.

Additional information related to the geophysical corrections used in the Sentinel-3 Hydro-Cryo Products is available in the dedicated Product Handbook.

References

Andersen, O. B., Rose, S. K., Abulaitijiang, A., Zhang, S., and Fleury, S.: The DTU21 global mean sea surface and first evaluation, Earth System Science Data, 15, 4065–4075, https://doi.org/10.5194/essd-15-4065-2023, 2023.

Cartwright, D. E. and Edden, A. C.: Corrected Tables of Tidal Harmonics, Geophysical Journal International, 33, 253–264, https://doi.org/10.1111/j.1365-246X.1973.tb03420.x, 1973.

Iijima, B. A., Harris, I. L., Ho, C. M., Lindqwister, U. J., Mannucci, A. J., Pi, X., Reyes, M. J., Sparks, L. C., and Wilson, B. D.: Automated daily process for global ionospheric total electron content maps and satellite ocean altimeter ionospheric calibration based on Global Positioning System data, Journal of Atmospheric and Solar-Terrestrial Physics, 61, 1205–1218, https://doi.org/10.1016/S1364-6826(99)00067-X, 1999.

Desai, S., Wahr, J., and Beckley, B.: Revisiting the pole tide for and from satellite altimetry, J Geod, 89, 1233–1243, https://doi.org/10.1007/s00190-015-0848-7, 2015.

Lyard, F., Lefevre, F., Letellier, T., and Francis, O.: Modelling the global ocean tides: modern insights from FES2004, Ocean Dynamics, 56, 394–415, https://doi.org/10.1007/s10236-006-0086-x, 2006.

Supplementary S2: Illustration of Hamming window and zero-padding in the SAR mode waveforms *(section 2.2: Delay-Doppler Processing)*

For Hydrology and Sea Ice Thematic Processors, the delay-Doppler is performed with Hamming window and zero-padding techniques. As mentioned in section 2.2, the zero-padding oversamples by a factor of 2 the Sentinel-3 SAR mode waveforms. This is illustrated in the Fig. S1 below, in the case of a Sentinel-3 peaky waveforms acquired over sea ice.

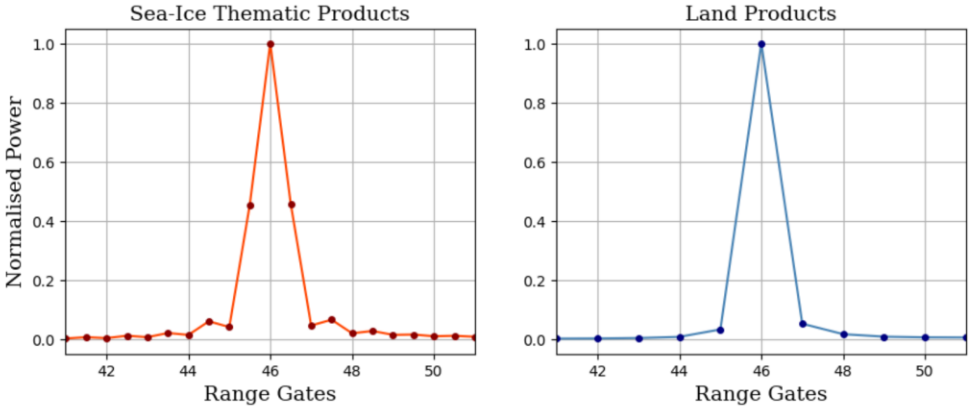

Figure S1: Illustration of zero-padding processing for a Sentinel-3A peaky SAR mode waveform acquired over sea ice, as generated by the Sea Ice Thematic (left) and the Land (right) Processors. With zero-padding the waveform leading edge is better sampled, allowing a finer estimation of the altimeter range.

The other new feature is the application of a Hamming weighting window. With no windowing applied, the azimuth impulse response is a "Sinc" squared function, with relatively strong secondary lobes, as mentioned section 2.2. In the case of specular echoes, energy spreads into the delay-Doppler map, in the azimuth direction, leading to spurious energy before to the main waveform leading edge (Fig. S2, top panel). The Hamming weighting window significantly reduces this undesired energy, that can generate retracking errors (Fig. S2, bottom panel).

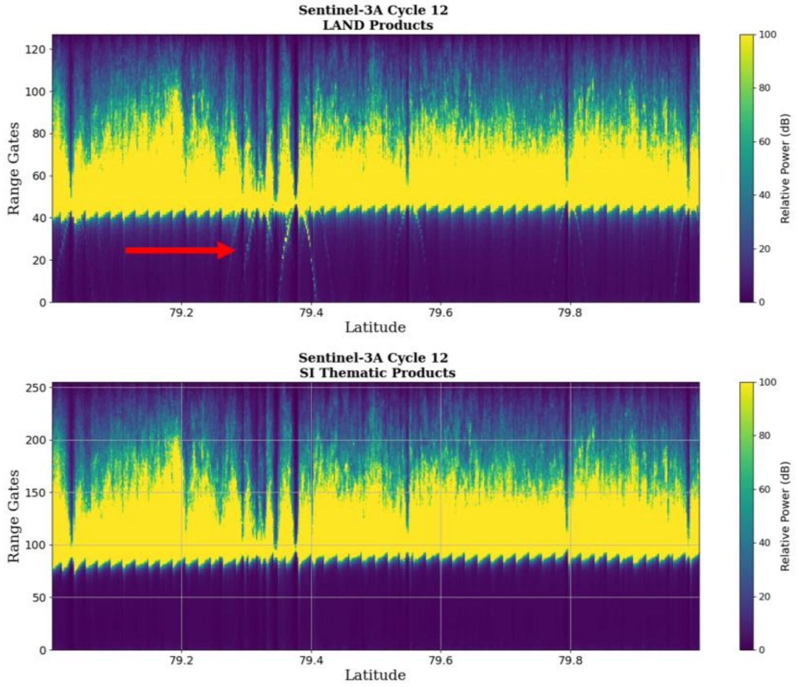

Figure S2: Illustration of the Hamming weighting window processing on Sentinel-3A radargrams over sea-ice. (top) SAR mode waveforms generated without Hamming window and zero-padding (bottom) SAR mode waveforms generated with the Thematic IPF, that includes Hamming window and zero-padding in the delay-Doppler processing.

Supplementary S3: Illustration of extended window processing *(section 2.2: Delay-Doppler Processing)*

As mentioned in the main text (section 2.2), the objective of the delay-Doppler with extended window is to recover additional useful backscattered energy in the delay-Doppler stack. In addition, the waveforms are repositioned in the 128-sample window analysis. The impact of this evolution on the SAR mode waveforms is illustrated in Fig. S3. The effect is mainly noticeable over the ice sheet margins, where surface slope and hm-km scale roughness increase. There are two main benefits for the SAR mode waveforms acquired over these regions. Firstly, the measurement Signal-to-Noise Ratio increases. Secondly, being recentred in the window analysis, the SAR mode waveforms can be more efficiently processed by a retracking algorithm. A quantification of the processing added value is provided in the main text, section 6.1.

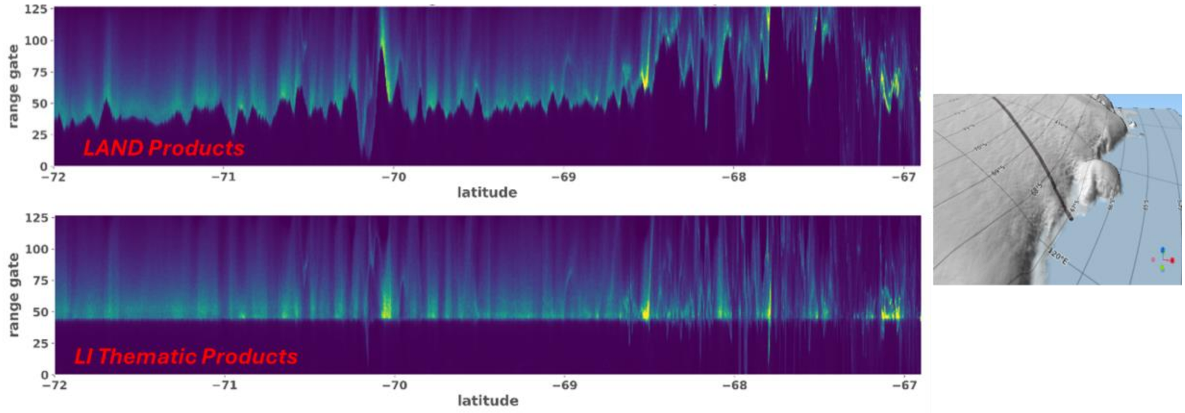

Figure S3: Sentinel-3A SAR mode waveforms acquired along a track portion over the Antarctic ice sheet, as generated by the previous Land Processor (top panel) and the new Land Ice Thematic Processor (bottom panel). The waveform power is normalised in amplitude using the maximum value in this track portion. The track location is displayed in the map in right figure.

Supplementary S4: Lake level processing *(Evaluation of Hydrology Thematic Products over lakes)*

To identify water level measurements from Sentinel-3, lake shoreline polygons from the SWOT (Surface Water and Ocean Topography) Prior Lake Data Base (Sheng et al., 2016), is applied. Only lakes with an area above 20 km^2 are included in the analysis, to ensure a substantial amount of data per lake. Additionally, the Global Surface Water Occurrence product (Pekel et al., 2016), is applied to refine the spatial selection of Sentinel-3 measurements, by using a 75% threshold in the water occurrence probability. The Sentinel-3 water surface height is extracted from the product variable "elevation_ocog_20_ku" and referenced with respect to the geoid model EGM-2008 (Pavlis et al., 2012) using the value provided in the Sentinel-3 Thematic Product. The R-package 'tsHydro' (https://github.com/cavios/tshydro) is employed to reconstruct the Sentinel-3 water level time series. The package is the implementation of a state-space model presented by Nielsen et al. (2015), where the process, accounting for the temporal correlation, is a Random Walk, and the observations are assumed to follow a mixture between Normal and Cauchy distributions, ensuring robustness. The mixture distribution is scaled to match the observation noise, and the scale parameter is used as a measure of the observation noise. Technically the observations are described as:

$$H_i = H_i^{true} + s\varepsilon_i$$

Here H_i is the water level of a single 20 Hz along-track observation, and H_i^{true} is the true water level (obtained from the process, here the Random Walk). ε_i is assumed to follow a mixture of a standardised Normal distribution and a standardised Cauchy distribution (10% Cauchy). In a similar standard normal model, the scale parameter is the standard deviation of the observation noise, but in a Cauchy mixture distribution the standard deviation is not defined but can be expressed via the scale s. Further details regarding the model are available in Nielsen et al. (2015). To minimise the effect of potential geoid errors a time series is reconstructed for each "lake-satellite track" combination, resulting in approximately 7,300 accepted time series distributed over 4,100 lakes.

References

Nielsen, K., Stenseng, L., Andersen, O. B., Villadsen, H., and Knudsen, P.: Validation of CryoSat-2 SAR mode based lake levels, Remote Sensing of Environment, 171, 162–170, https://doi.org/10.1016/j.rse.2015.10.023, 2015.

Pavlis, N. K., Holmes, S. A., Kenyon, S. C., and Factor, J. K.: The development and evaluation of the Earth Gravitational Model 2008 (EGM2008), J. Geophys. Res., 117, 2011JB008916, https://doi.org/10.1029/2011JB008916, 2012.

Pekel, J.-F., Cottam, A., Gorelick, N., and Belward, A. S.: High-resolution mapping of global surface water and its long-term changes, Nature, 540, 418–422, https://doi.org/10.1038/nature20584, 2016.

Sheng, Y., Song, C., Wang, J., Lyons, E. A., Knox, B. R., Cox, J. S., and Gao, F.: Representative lake water extent mapping at continental scales using multi-temporal Landsat-8 imagery, Remote Sensing of Environment, 185, 129–141, https://doi.org/10.1016/j.rse.2015.12.041, 2016.

Supplementary S5: Range noise level analysis (*Evaluation of Hydrology Thematic Products over Rivers)*

In this analysis, we propose a computation of the 20 Hz noise level for the altimeter range estimated over inland waters. The noise level is calculated based on comparisons of successive 20 Hz Sentinel-3 estimates (*Orbit* and *Range* for "*alt_20_ku*" and "*range_ocog_20_ku*" product variables, respectively). It must be noted that the geophysical corrections are not applied to the "*range_ocog_20_ku*" parameter. Each section of measurements over a given lake is called a "transect". For a 20 Hz measurement *n,* of a given transect *t,* the value $\Delta(Orbit - Range)_{tn}$ is computed such as:

$$\Delta(Orbit - Range)_{tn} = (Orbit - Range)_{n+1} - (Orbit - Range)_n$$

where *n* and *n+1* are two consecutive Sentinel-3 measurements of the transect *t*. For a transect composed of at least $N \geq 2$ measurements, $N-1$ $\Delta(Orbit - Range)_{tn}$ values are computed.

This computation was done for all the Sentinel-3A and Sentinel-3B transects located over the lakes included in the HydroLakes database (Messager et al., 2016). We analysed the measurements over small to moderate size lakes only, by selecting those with an area between 1 km^2 and 5 km^2. The objective is to restrain the analysis to SAR mode waveforms with high peakiness values, for which the evolutions made on the delay-Doppler processing are assumed to be valuable. The measurements located above 40°N and below 40°S are removed, to avoid the inclusion of frozen lakes. For each orbit cycle, there are in average 1555 $\Delta(Orbit - Range)_{tn}$ values, located in average over 674 lakes that were combined to obtain statistic distributions. A gaussian curve is fitted on each cyclic distribution, and an average range noise level value is estimated per orbit cycle, by taking the standard deviation of this curve. This assessment was performed over the Sentinel-3A and Sentinel-3B complete available data set, for both the former Land and new Hydrology Thematic Products. In the left panels of Fig. S4, examples of $\Delta(Orbit - Range)_{tn}$ distributions are displayed for cycle 101 of Sentinel-3A and cycle 83 of Sentinel-3B. In the right panel of Fig. S4, temporal evolution of the average noise range level is represented for both the former Land and new Hydrology Thematic Products.

We observe that the range noise level estimated at 20 Hz rate is stable throughout cycles, for both the former Land and new Hydrology Thematic Products. When averaged over the time series, noise level mean values are 20.1 cm and 5.7 cm, for Land and Hydrology Thematic Products, respectively. Therefore, the Thematic Products provide a significant noise level reduction, over the relatively small water bodies selected, by a factor of around 3.

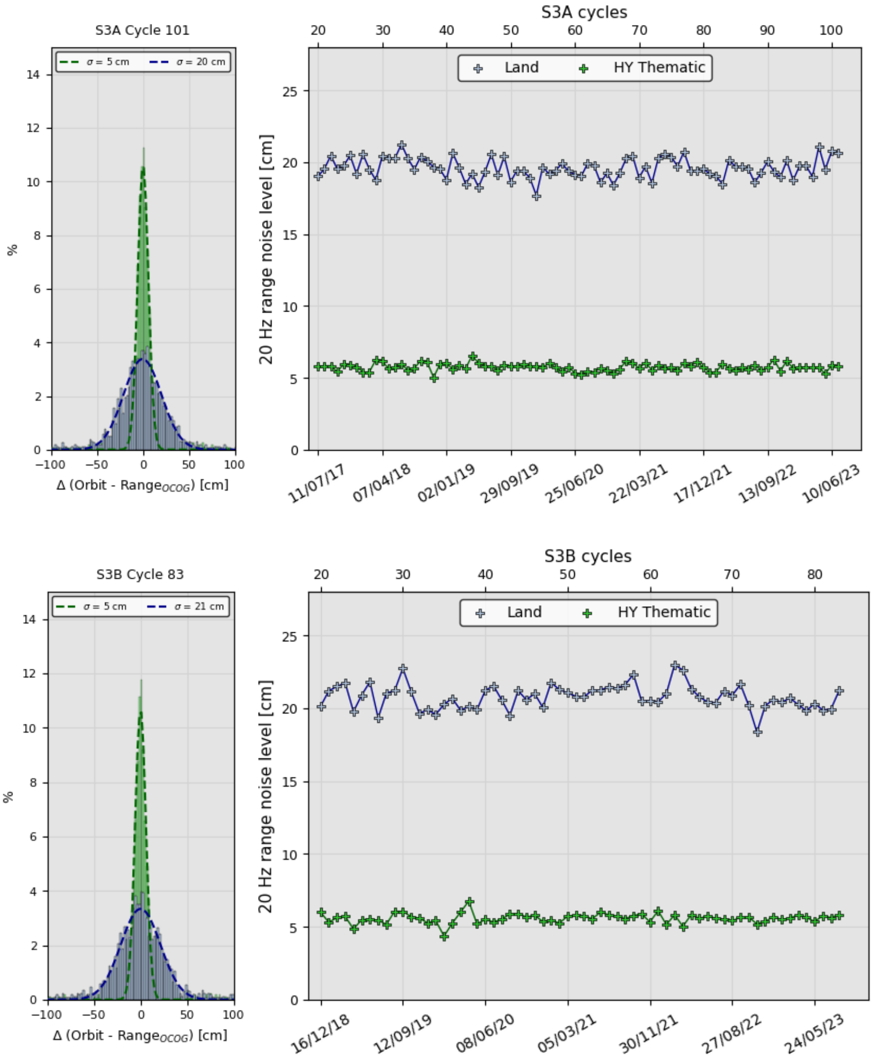

Figure 1S4: Left: examples of Δ(Orbit – Range) distributions for Land (blue) and Hydrology Thematic (green) Products for the cycle 83 and 101 of S3B (top) and S3A (bottom) respectively. Standard deviations of these distributions are the estimated values of the range noise level. Right: evolution of the average range noise level over time for S3B (top) and S3A (bottom)

References

Messager, M. L., Lehner, B., Grill, G., Nedeva, I., and Schmitt, O.: Estimating the volume and age of water stored in global lakes using a geo-statistical approach, Nat Commun, 7, 13603, https://doi.org/10.1038/ncomms13603, 2016.

Supplementary S6: Illustration of Water Surface Height time series (*Evaluation of Hydrology Thematic Products over Rivers*)

In this supplementary, we illustrate the approach detailed in section 4.2 which is based on comparisons between in-situ and altimeter WSH timeseries. For each in-situ station, WSH measurements of Sentinel-3A and Sentinel-3B are compared to WSH measurements from the in-situ station, and the relative bias and MAD are computed to quantify the accuracy and precision, respectively. In this example for one in-situ station, the map shows the selected Sentinel-3 measurements around the virtual station, and the Sentinel-3 WSH timeseries computed based on the previous approach are represented for both previous Land and Hydrology Thematic Products.

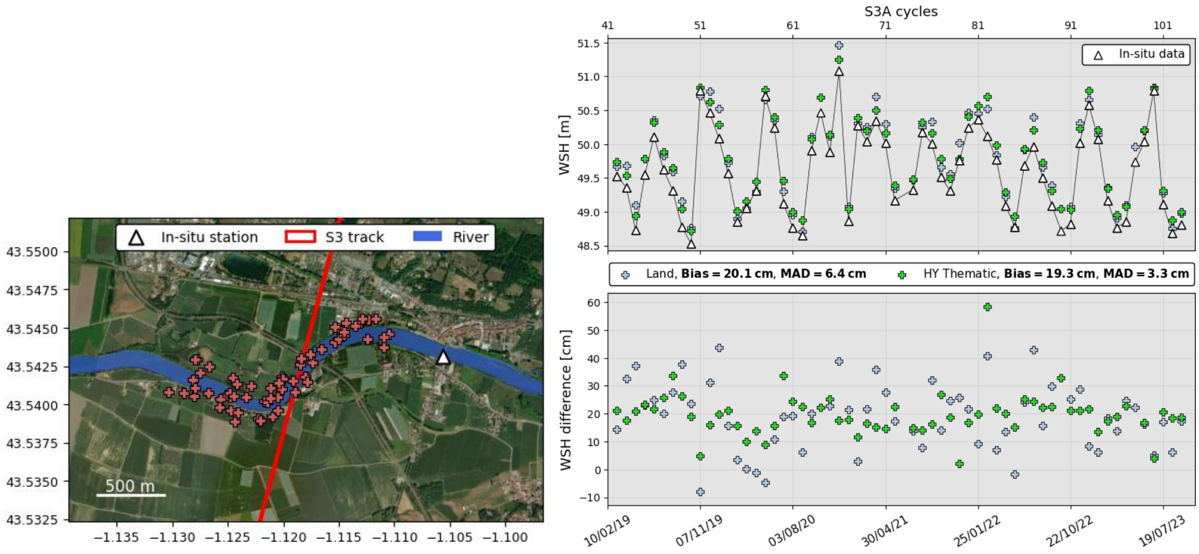

Figure S5: Left: Map of "Les Gaves Réunis" river (blue area) with Sentinel-3A ground track (red line), the in-situ station (white triangle), and the Sentinel-3A measurements (colored crosses) selected at the nadir location. Right: (Top) Timeseries of WSH from in-situ measurements (white triangles), S3A measurements from the Land products (blue crosses) and Hydrology Thematic Products (green crosses). (Bottom) Timeseries of the WSH difference

Supplementary S7: Difference in the sea ice type classification performed in the ground segment processing, between former Land and new Thematic Products *(Evaluation of Sea Ice Thematic Products - availability of freeboard measurements)*

In the Sea Ice Thematic IPF, a critical step towards the computation of the radar freeboard is the classification of measurements either as “floe” (i.e. sea ice), “lead” or “open ocean”. This is performed by applying a set of thresholds to the waveform peakiness parameter, and the sea ice concentration, which is extracted from OSI-SAF 430 product. Data measurements that do not meet any of the three criteria are considered as “unclassified”. Thanks to the Hamming and zero-padding techniques, the Sea Ice Thematic SAR waveforms are now free from off-nadir spurious energy, and better sampled. This induces a reduction of the number of “unclassified” measurements, and more importantly, an increase of the number of measurements identified as leads and sea ice floes that are used to compute the radar freeboard.

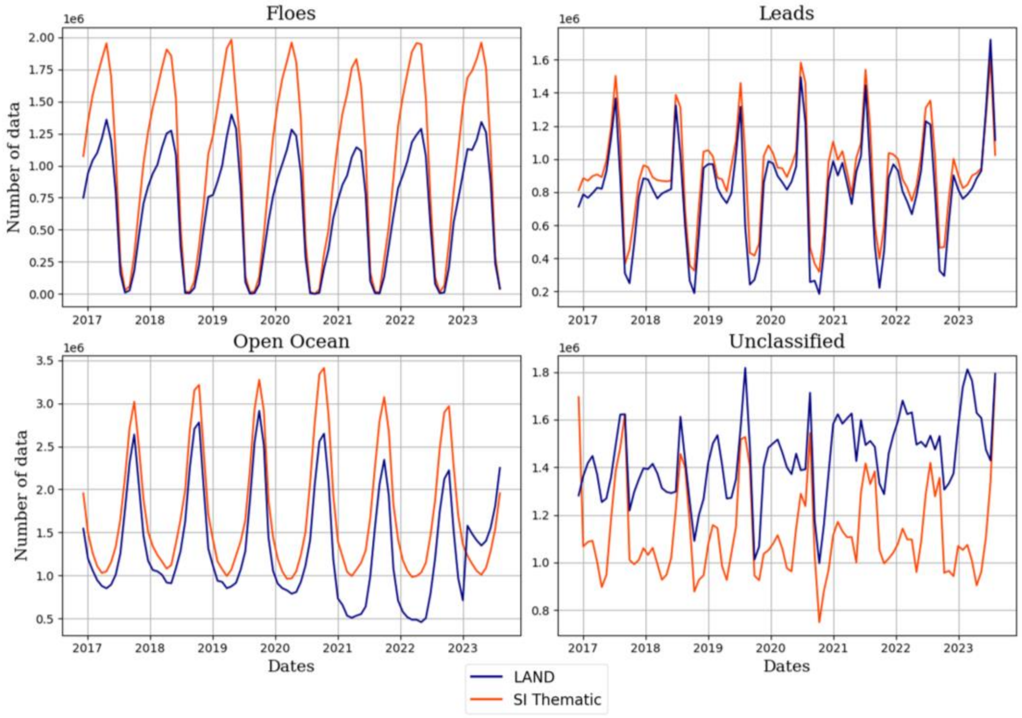

Figure S6: Time series of the number of measurements classified as sea ice floes (top left), leads (top right), open ocean (bottom left) and unclassified (bottom right) as discriminated by the surface classification flag ‘surf_type_class_20_ku’, for the former LAND products in blue and the SI thematic products in red. Here for Sentinel-3A Arctic.

Supplementary S8: Signal to Noise Ration (SNR) calculation over ice sheets *(Evaluation of Land Ice Thematic Products – data coverage improvement)*

A backscatter coefficient (Sigma-0) is computed for the ice sheet measurements (dB unit), according to:

$$Sigma0 = 10 * log_{10}(A_{max}) + S_f + S_b + C_o$$

where A_{max} is the maximum amplitude of the Sentinel-3 SAR mode waveform,
S_f is a scaling factor, read in the Land Ice Thematic Product (variable "*scale_factor_20_ku*"),
S_b is a systematic bias, applied in the Sentinel-3 IPF, equal to -0.65 dB,
C_o a calibration offset, calculated to roughly align this Sigma-0 with the one from the physical SAMOSA retracker (calibration performed over the open ocean), equal to -18 dB

The measurement is considered as "low SNR" if the Sigma-0 is lower than a -12 dB threshold. Below this threshold we noticed that approximately less than 66% of the SAR mode waveforms do not contain a clear energy peak, as displayed in Fig. S7. We also observed a relative strong degradation of the performances below this threshold. For the former Land Products and Land Ice Thematic Products, low SNR measurements respectively represent 3.4% and 1.7% of the data set. The lower ratio observed for the Land Ice Thematic Product is explained by the positive effect of the extended window processing.

It must be noted that the Sigma-0 estimated with the former Land and Land Ice Thematic Products remains at a similar level over the ice sheet interior, as expected. The Sigma-0 was estimated respectively at 6.89 dB (Land) and 6.76 dB (Land Ice Thematic) over lake Vostok, with Sentinel-3A and Sentinel-3B measurements acquired from 1 June 2022 to 30 August 2022 (over 3 orbit cycles of 27 days).

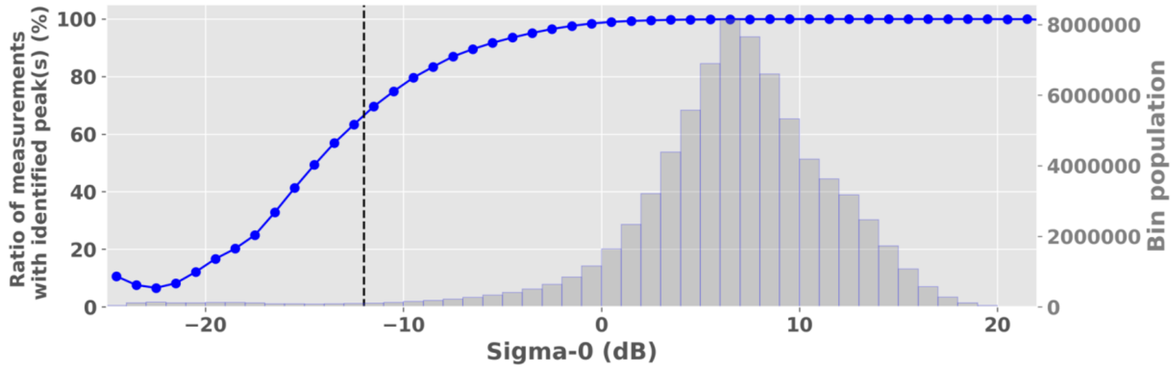

Figure S7: Ratio of Sentinel-3 measurements for which a clear energy peak is measured in the SAR mode waveforms, as a function of the Sigma-0. The peak detection is performed with the Leading Edge Detection algorithm (Aublanc et al., 2021). Grey bars display the number of measurements for each bin interval.

Supplementary S9: Performance comparison between former Land and new Land Ice Thematic Products *(Evaluation of Land Ice Thematic Products – accuracy and precision compared to ICESat-2 ATL06)*

In Fig. S8, the median bias and MAD of the elevation differences between nearly co-located Sentinel-3 and ICESat-2 measurements are represented as a function of the surface slope. The statistics are produced with the same data and methods as described in section 6.2. The results obtained with the Land Ice Thematic Products are the same ones as reported in Fig. 9 in the main text.

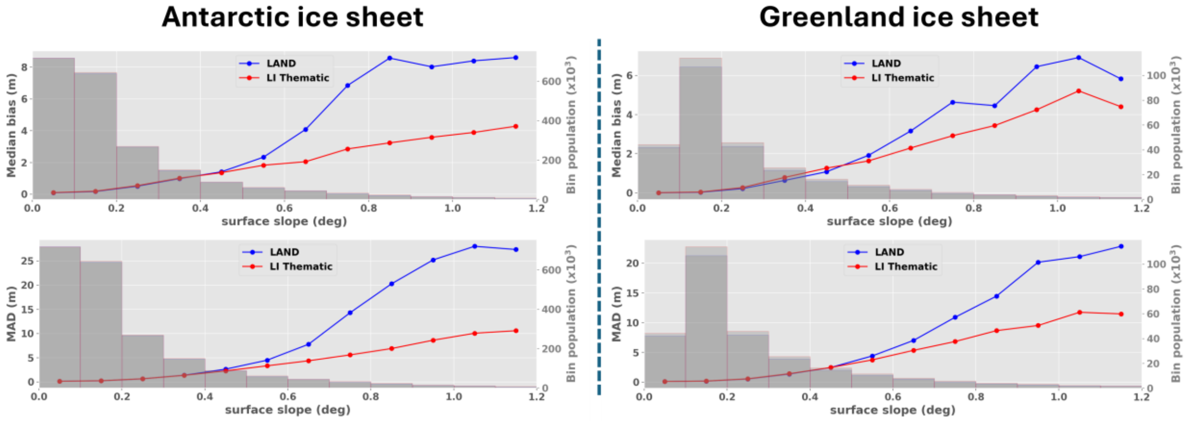

Figure S8: Median bias (top) and Median Absolute Deviation (bottom) between Sentinel-3 and ICESat-2 ATL06 colocated elevations represented as a function of surface slope, for the former Land (blue) and Land Ice Thematic Products (red). The statistics are computed over the Antarctic (left) and Greenland (right) ice sheets. Elevation differences are computed as Sentinel-3 – ICESat-2. Grey bars display the number of colocated measurements for each slope interval.

As it can be deduced from Fig. S8, an improvement is observed over the ice sheet margins with the Land Ice Thematic Products. For example, where slope is above 0.5° the median bias and MAD relatives to ICESat-2 ATL06 are respectively reduced by approximately 41% and 44% over the Antarctic ice sheets, respectively. The same ratios are 17% and 28% over the Greenland ice sheets.
